# Human Umbilical Cord-Based Therapeutics: Stem Cells and Blood Derivatives for Female Reproductive Medicine

**DOI:** 10.3390/ijms232415942

**Published:** 2022-12-14

**Authors:** Adolfo Rodríguez-Eguren, María Gómez-Álvarez, Emilio Francés-Herrero, Mónica Romeu, Hortensia Ferrero, Emre Seli, Irene Cervelló

**Affiliations:** 1 IVI Foundation, Health Research Institute La Fe, 46026 Valencia, Spain; 2 Department of Obstetrics, Gynecology and Reproductive Sciences, Yale School of Medicine, New Haven, CT 05610, USA; 3 Department of Pediatrics, Obstetrics and Gynecology, School of Medicine, University of Valencia, 46010 Valencia, Spain; 4 Gynecological Service, Consortium General University Hospital of Valencia, 46014 Valencia, Spain; 5 IVIRMA New Jersey, Basking Ridge, NJ 07920, USA

**Keywords:** umbilical cord, female reproduction, umbilical cord mesenchymal stem cells, stem cell therapy, platelet-rich plasma, exosomes, growth factors, ovary, endometrium

## Abstract

There are several conditions that lead to female infertility, where traditional or conventional treatments have limited efficacy. In these challenging scenarios, stem cell (SC) therapies have been investigated as alternative treatment strategies. Human umbilical cord (hUC) mesenchymal stem cells (hUC-MSC), along with their secreted paracrine factors, extracts, and biomolecules, have emerged as promising therapeutic alternatives in regenerative medicine, due to their remarkable potential to promote anti-inflammatory and regenerative processes more efficiently than other autologous treatments. Similarly, hUC blood derivatives, such as platelet-rich plasma (PRP), or isolated plasma elements, such as growth factors, have also demonstrated potential. This literature review aims to summarize the recent therapeutic advances based on hUC-MSCs, hUC blood, and/or other plasma derivatives (e.g., extracellular vesicles, hUC-PRP, and growth factors) in the context of female reproductive medicine. We present an in-depth analysis of the principal molecules mediating tissue regeneration, compiling the application of these therapies in preclinical and clinical studies, within the context of the human reproductive tract. Despite the recent advances in bioengineering strategies that sustain delivery and amplify the scope of the therapeutic benefits, further clinical trials are required prior to the wide implementation of these alternative therapies in reproductive medicine.

## 1. Introduction

Infertility is a disease of the male or female reproductive system, defined by the World Health Organization as the failure to achieve a pregnancy, after at least 12 months of regular unprotected sexual intercourse [1]. Specifically, female infertility can be caused by various disorders of the reproductive system, including premature ovarian insufficiency (POI), polycystic ovary syndrome (PCOS), endometriosis, Asherman’s syndrome (AS), or endometrial atrophy (EA), among others.

Several preclinical studies and clinical trials have been conducted to evaluate the use of stem cells (SCs), and/or their derivatives, as an alternative strategy for treating different reproductive disorders that lead to infertility [2,3,4]. Stem cells are undifferentiated cells, with the potential to perpetuate self-renewal, for a long period of time. They can divide into identical SCs (via symmetrical division), or, under certain physiologic or experimental stimulus, give rise to differentiated (mature) cells with particular functions (via asymmetric division or tissue-specific differentiation processes) [5]. Several studies have focused on mesenchymal stem cells (MSCs) for experimental approaches to treat infertility [6,7,8,9,10,11,12,13,14,15,16]. In this context, there are various sources of human MSCs used in therapeutic approaches for regenerative medicine, such as the bone marrow, adipose tissue, menstrual blood, salivary glands, dental pulp, amniotic fluid, placental tissue, and umbilical cord (UC), the latter being the focus of this review [17,18].

Until the 1990s, the human UC and its blood derivatives were considered medical waste. However, the collection of human UC-MSCs (hUC-MSC) is considered non-invasive, and thus not encumbered with ethical problems. Remarkably, hUC-MSCs have some exceptional characteristics, including rapid self-renewal, low oncogenicity, and poor immunogenicity (due to their low expression of the major histocompatibility complex (MHC) class I and class II proteins), making them an important source of SCs for allogeneic transplantation therapy without immune rejection [19,20]. Moreover, the cell source is heterologous, its procurement is non-invasive and is not associated with any comorbidity for the patient. Considering these features, hUC-MSCs are preferentially used over other sources of MSCs, for auto- and allo-transplantation. Finally, different methods have been developed to isolate hUC-MSCs (i.e., such as from Wharton’s jelly, arteries or veins) [20,21,22,23]. To date, cell therapy based on hUC-MSCs has been applied in a multitude of medical disciplines, for regenerative and immunomodulatory purposes [20,24,25,26,27,28,29], with gynecology being no exception [30,31,32,33,34,35,36,37,38,39,40]. Nevertheless, many concerns remain regarding their use and long-term safety, and most of the treatments developed thus far are still considered invasive and experimental [41,42].

In this regard, new strategies have been investigated. In particular, the synthesis and secretion of chemokines, growth factors (GFs), blood-extracts, biomolecules, and hormones by hUC-MSCs affects the adjacent cells through paracrine signaling. These components play important roles in angiogenesis, anti-inflammation, immunomodulation, anti-apoptosis, and anti-fibrosis, thereby contributing to the regeneration of injured tissues [43]. For example, microvesicles and exosomes are extracellular vesicles (EVs) that carry membrane and cytoplasmic constituents of intracellular origin, and can transfer proteins, messenger RNAs, microRNAs, and bioactive lipids to target cells (with specific surface receptors) to affect their phenotype and function [44,45]. Based on this premise, some groups have explored using hUC-MSC-derived conditioned media, and hUC-MSC-secreted vesicles, rather than the hUC-MSCs directly, to examine the indirect therapeutic effects on damaged tissues [43,46]. Notably, the microvesicles and exosomes released from MSCs exerted a protective effect on reproductive tissues, and rescued ovarian function, in vitro and in vivo [47,48,49].

Overall, hUC offers a plethora of valuable byproducts for regenerative medicine, including whole hUC blood and its derivatives (i.e., serum, plasma, endothelial progenitor cells, MSCs, and hematopoietic cells). Although hUC blood is mainly used to treat blood disorders via hematopoietic SC transplantation, hUC-MSCs have garnered the most attention for therapeutic purposes [50,51]. Recently, platelet-rich plasma (PRP), a blood-derived product that can be prepared commercially and contains five to ten times the density of platelets than normal blood [52], has also emerged as a promising regenerative mediator. Interestingly, studies analyzing human umbilical cord platelet-rich plasma (hUC-PRP) reported that it produced a higher concentration of GFs, chemokines, and cytokines than adult peripheral blood PRP, and contributed to tissue regeneration through enhancement of vascular endothelial growth factor (VEGF) and platelet-derived growth factor (PDGF) [53,54,55,56]. So far, hUC-PRP has shown promising preclinical results, for epidermolysis bullosa [57,58], oral mucositis [59], and foot ulcers associated with diabetes [60], and reversing chemotherapy-induced ovarian damage [61].

Overall, UC blood offers numerous biological agents/products with high regenerative and immunomodulatory potential. This bibliographic review aims to compile the most relevant studies in the context of female reproduction, highlighting the advances and the emerging therapeutic strategies.

## 2. Human Umbilical Cord: Composition and Potential Mechanisms of Action

### 2.1. The Cellular Components

The hUC blood contains different cell types, including hUC-derived unrestricted somatic SCs, embryonic-like SCs, multipotent progenitor cells, and endothelial progenitor cells [62]. However, the most studied are the hUC-MSCs, which exert potent immunosuppressive and anti-inflammatory effects, as demonstrated with cell-based therapies for heart injury [63,64], retinitis pigmentosa [65], type II diabetes [66], and female pathologies [67,68], among others.

**Figure 1:**
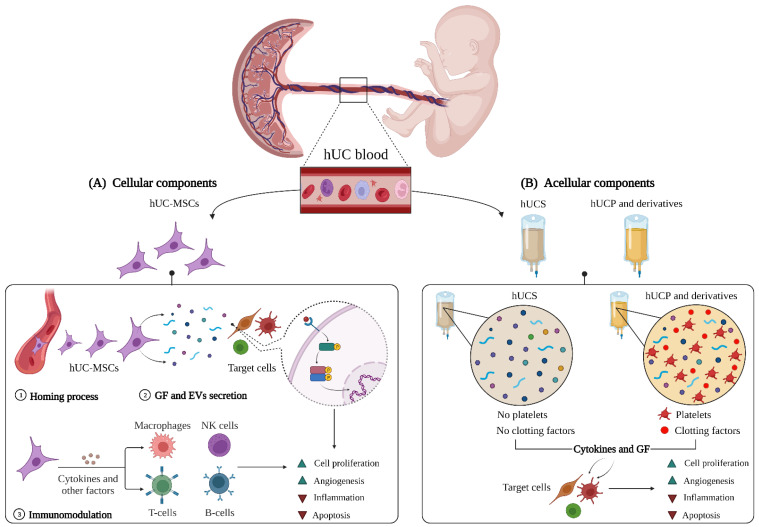
The hUC blood composition. This illustration depicts the roles of the (**A**) cellular and (**B**) acellular components, in tissue regeneration processes. EVs, extracellular vesicles; GF, growth factors; hUC, human umbilical cord; hUC-MSCs, human umbilical cord mesenchymal stem cells; hUCP, human umbilical cord plasma; hUCS, human umbilical cord serum; and NK cells, natural killer cells.

All these cellular components, particularly hUC-MSCs, utilize three processes during cell therapy (Figure 1A): (i) homing (the process of migrating to and settling in the damaged tissue), (ii) secretion of GFs and EVs, and (iii) immunomodulation. Briefly, hUC-MSCs colonize the injury site, and participate in cell proliferation and angiogenesis, through the release of paracrine factors (e.g., cytokines, GFs, and EVs), while simultaneously modulating the immune system to control inflammation and promote tissue regeneration [62]. To elucidate the complexities of these processes, each one is described in detail below, to elaborate on relevant cell functions and/or hUC blood factors.

#### 2.1.1. The Homing Process

When tissue integrity is compromised, the surrounding cells release inflammatory molecules and chemokines, producing a concentration gradient of ligands that recruits innate immune cells, via a phenomenon known as chemotaxis. Stem cells are attracted in response to this chemical stimulus and migrate directly to the site of damage to initiate specific regenerative functions. This homing effect is made possible by the adhesive junctions between SCs and the vascular endothelium at the target tissue. Specifically, the cell-rolling interactions are mediated by the homing receptors expressed on the SCs and their cognate endothelial co-receptors. The activation of integrins anchor the engrafted cells for extravasation [62,69]. To achieve this cell mobilization, MSCs express the most common immune-cell-homing chemokine receptors, including C-X-C chemokine receptor type 4 (CXCR4) and C-C chemokine receptor (CCR), which interact with the potent chemoattractants that are upregulated at the injury site, such as stromal-cell-derived factor 1 (SDF1) and monocyte-chemotactic protein 3 (MCP3) [70,71].

#### 2.1.2. Secretion of Paracrine Factors

After homing, SCs secrete cytokines, biomolecules, trophic and growth factors. These molecules impact target cells by modulating inflammation/apoptosis, triggering progenitor cell proliferation, and stimulating tissue repair to provide favorable conditions for cell survival. Indeed, GFs and EVs are regarded as the two main components implicated in SC paracrine signaling [62].

##### Growth Factors

Hundreds of GFs (including VEGF, nerve growth factor (NGF), endothelial growth factor (EGF), fibroblast growth factor (FGF), hepatocyte growth factor (HGF), and transforming growth factor beta (TGFβ)) and cytokines (such as interleukins (ILs), granulocyte colony-stimulating factor (G-CSF), and granulocyte–macrophage colony-stimulating factor (GM-CSF)) are secreted by hUC-MSCs, after fusion of secretory granules with the plasma membrane, to immediately begin coordinating local tissue regeneration [72,73] (Table 1). These factors interact with membrane receptors on adjacent recipient cells [62], to mediate angiogenesis (in particular VEGF), cell chemotaxis (mainly driven by specific chemokines), anti-apoptotic effects (particularly G-CSF and NGF), proliferation and immunomodulation [74]. Interestingly, adult bone marrow MSCs and hUC-MSCs have comparable cytokine expression profiles, which translate into similar biological potential [75].

The paracrine action of the hUC-MSCs has previously been described in various pathologies, such as renal injury [76,77,78], brain damage [79,80], cardiac injury [64,81], and female reproductive pathologies. For example, G-CSF has shown protective effects in ovarian damage by activating the PI3K/AKT pathway to promote cell survival and proliferation of primordial follicles and granulosa cells [82,83]. Notably, this signaling pathway is co-activated by NGF, which, in turn, signals through the tropomyosin receptor kinase A (TrkA) receptor, contributing to these downstream cellular responses [84]. Alternatively, TGFβ binds to its cognate receptors on the oocyte surface and inhibits follicular cell apoptosis in ovarian failure [85]. Taken together, these findings suggest that each GF modulates a particular molecular pathway, contributing to a complex network of cell processes, that ultimately produce a tailored response in the target tissue. Remarkably, many of these factors appear to act synergistically [84], which partially justifies the difficulty of elucidating the individual roles of each GF secreted by hUC-MSCs.

##### Extracellular Vesicles

The therapeutic benefits produced by the EVs derived from hUC-MSCs depend on their mechanism of action and the pathophysiology of each disease. For example, in rats with acute kidney injury, treatment with EVs downregulated pathways activated by p38 mitogen-activated protein kinase (MAPK), which consequently decreased the caspase 3 protein expression and favored renal cell survival. In this context, EVs also promoted cell proliferation, through activation of the extracellular-signal-regulated kinase (ERK) 1/2 pathway, reducing creatinine levels, kidney tubule necrosis, apoptosis, and oxidative stress in vivo [86]. Meanwhile, treating liver fibrosis (characterized by the excessive production and deposit of collagen resulting from tissue repair process) with EVs reverted the conversion of hepatocytes to fibroblasts (that occurs through the epithelial-to-mesenchymal transition (EMT)), both in vitro and in vivo. Specifically, the molecules carried by the EVs induced downregulation of TGFβ, which inhibited SMAD family member 2 (Smad2) phosphorylation, that is essential for the transcription of genes responsible for the EMT [87]. Interestingly, female disorders such as intrauterine adhesions [88] or PCOS [89] also benefited from EV treatment both in vivo and in vitro. In both these cases, the therapeutic effect of the EVs was directed by the post-transcriptional modifications produced by the micro-RNAs, that induced M2 macrophage polarization and inhibited the expression of pro-inflammatory molecules [88,89].

While small non-coding RNAs are usually transported by EVs, they can also be secreted directly by the hUC-MSCs to modulate other signaling cascades [90,91,92,93,94]. Indeed, the upregulation of miR-455-5p and miR-330-5p in hUC-MSCs has been associated with different mechanisms that attenuate endometrial injury and promote repair of murine damaged endometrium. In particular, miR-455-5p regulates the suppressor of cytokine signaling 3 (SOCS3)-mediated Janus kinase (JAK)/signal transducer and activator of transcription 3 (STAT3) signaling pathway [90], whereas miR-330-5p stabilizes mitochondrial metabolism through pyruvate dehydrogenase kinase 2 (PDK2) signaling [93].

#### 2.1.3. Immunomodulation

Since the hUC-MSCs scarcely express MHC class II proteins and co-stimulatory molecules, they are considered immunoprivileged cells [29]. These cells can suppress the immune system by inhibiting the physiological functions of T-, B-, and natural killer (NK) cells (e.g., cell proliferation, production of GFs, and cytotoxicity), through cell–cell contact, and the secretion of paracrine factors. Specifically, during adaptive immune responses, hUC-MSCs restrained the activation and proliferation of cluster of differentiation (CD) 4 (CD4)+, CD8+, CD2+, and CD3+ T-cells subpopulations, while stimulating regulatory T-cells [95]. In addition, hUC-MSCs significantly suppressed the proliferation, differentiation, and immunoglobulin secretion of B-cells, in vitro, by modulating the MAPK signaling pathway [96]. Regarding the innate immune response, hUC-MSCs suppressed NK cells by secreting prostaglandin-E2 [97], and converted monocytes and dendritic cells to an immature state [29]. Although hUC-MSC-mediated immunosuppression has been described in many studies [29,95,96,98,99], its complexities merit further investigation. Furthermore, the hUC-MSCs’ immune modulating effects will notably depend on the ratio of hUC-MSCs and immune cells (especially B-cells), immune cell activation state and maturation stage. In this regard, the microenvironment is crucial for determining the immunomodulatory effects of the hUC-MSCs [96].

Notably, recent studies proposed using the factors secreted by hUC-SCs, rather than cell transplantation, for regenerative medicine [100,101,102,103]. The secreted GFs are referred to as the secretome of the hUC-SCs, can be isolated from culture media, and induce biological effects similar to SC therapy. The advantages of using this conditioned culture medium include that it can be manufactured more easily, is acellular, immunotolerated, and does not promote tumorigenesis [104]. On the other hand, several authors have isolated EVs by ultracentrifugation [105] and studied the specific effects of SC-derived EVs [81,87,89,106,107,108,109,110], including the re-epithelialization of cutaneous wounds [111], muscle regeneration [112], and modulation of immune system [113], among others. Nevertheless, it is still necessary to compare the efficacy and potency of conditioned media and EV-based therapies with SC transplantation, in controlled clinical trials.

### 2.2. The Acellular Fractions

The postulate that tissue repair by hUC-MSCs is mediated via paracrine signaling suggests that the GFs and cytokines these cells produce are sufficient to activate their exceptional regeneration abilities and provides an alternative approach for using hUC blood derivatives in regenerative medicine.

In this context, recent studies have focused on using the acellular fractions of the hUC blood, namely the human umbilical cord serum (hUCS) and plasma (hUCP) (Figure 1B). Since their GFs and cytokines produced comparable anti-inflammatory, angiogenic, and anti-apoptotic effects, the main difference between both biofluids is the absence (hUCS) or presence (hUCP) of clotting factors [114,115]. In addition, since hUCS and hUCP resemble the ‘culture medium’ of hUC blood cells, it is possible that they contain some GFs secreted by hUC-MSCs, and therefore partly contribute to tissue regeneration processes [104].

#### 2.2.1. Human Umbilical Cord Serum

The hUCS is the non-cellular supernatant obtained when whole hUC blood clots, and is rich in cytokines and GFs with relevant biological properties, such as many ILs, G-CSF, GM-CSF, FGF, NGF, tumor necrosis factor beta, and VEGF, among others [114,115] (Table 1). Compared to hUCP, the hUCS is distinguished by a higher concentration of VEGF, which has been associated with pronounced angiogenic and anti-apoptotic effects, via PI3K/AKT and MAPK signaling pathways. However, the elevated secretory activity of the hUC blood cells, contributing to the composition of the hUCS during clotting, may also lead to this concentration difference [115].

The hUCS has been utilized as a substitute for fetal bovine serum, due to its equal or even greater culture potential with lower immunogenicity [114]. In fact, many studies have demonstrated its versatility for expanding cultures of different cell types, ranging from multipotent mesenchymal stromal cells [116] to epithelial cells [117,118] and oocytes for assisted reproductive techniques [119]. Furthermore, hUCS has shown remarkable efficacy in experimental and clinical settings, particularly in ocular injury [120,121,122,123,124]. In these cases, it provided neuroprotective action on the optic nerve, by significantly decreasing the presence of pro-inflammatory cytokines [125].

#### 2.2.2. Human Umbilical Cord Plasma

In contrast to hUCS, isolating hUCP requires the addition of an anticoagulant to prevent the platelet-mediated clotting process. hUCP is also enriched with powerful stimulators of regeneration and resident progenitor cells, however its concentration of pro-inflammatory cytokines is minimal [115]. Following centrifugation, the platelet content of hUCP can be concentrated up to 4–5 times to obtain hUC-PRP. Once activated, platelets change shape and release the contents of their alpha granules into their surroundings to chemoattract host cells that repair the tissue injury [126,127]. Next, platelets secrete specific proteins (e.g., PDGF, TGFβ, VEGF, and EGF) that initiate intracellular signaling cascades which produce specific cell responses (Table 1). Target cells include fibroblasts, bone marrow-derived SCs, and pre-osteoblasts (respectively, representing long-term healing, bone regeneration, and bone remodeling) along with immune cells (i.e., macrophages, which, in response to hUC-PRP, have decreased inflammation in numerous diseases [128]). Remarkably, while adult human PRP has been used extensively in many therapeutic areas [129,130,131,132,133,134,135,136], clinical application of hUC-PRP remains experimental [137,138,139].

**Table 1:** Main growth factors secreted by hUC-MSCs and found in the acellular components of hUC blood. For each growth factor, this table lists the most prominent signaling pathways, biological functions, and therapeutic applications reported in the literature in reproductive medicine and other medical fields.

Type of Factor	Signaling Pathways	Biological Functions	Therapeutic Applications	References
VEGF	PI3K/AKT Ras/MAPKs Src/FAK	✓ Angiogenesis; ✓ Vessel permeability; ✓ Cell proliferation; ✓ Cell motility; ✓ Immune cell chemotaxis.	Reproductive medicine: EA, IUA, POI Other fields: Neurodegenerative diseases, chronic diabetic wounds, neuropathic pain	[54,110,139,140]
NGF	PI3K/AKT Ras/MAPKs PLC-ϒ JNK	✓ Growth and survival of sensory nerves; ✓ Inhibition of apoptosis; ✓ Cell proliferation.	Reproductive medicine: POI Other fields: Brain and nerve injury, myocardial infarction, diabetic cystopathy	[84,141,142,143,144]
EGF	PI3K/AKT Ras/MAPKs JAK/STATs	✓ Mitosis, differentiation, and chemotaxis in epithelial and mesenchymal cells; ✓ Secretion of cytokines.	Reproductive medicine: IUA, POI Other fields: Dry eye syndrome, atopic dermatitis	[124,145]
FGF	PI3K/AKT Ras/MAPKs	✓ Proliferation, growth, and differentiation of mesenchymal cells, chondrocytes, and osteoblasts; ✓ Angiogenesis.	Reproductive medicine: EA, IUA, vaginal reconstruction Other fields: Autoimmune encephalitis, chronic diabetic wounds, amyotrophic sclerosis, osteoarthritis	[144,146,147,148]
HGF	PI3K/AKT Ras/MAPKs JAK/STATs	✓ Regulation of cell migration; ✓ Cell growth; ✓ Fibrosis inhibition; ✓ Regulation of wound healing; ✓ Immune modulation.	Reproductive medicine: POI Other fields: Parkinson’s disease, cardiopathies, liver fibrosis	[149,150,151,152]
G-CSF	PI3K/AKT Ras/MAPKs JAK/STATs	✓ Cell survival and proliferation; ✓ Enhancement of mature neutrophil function.	Reproductive medicine: POI, recurrent implantation failure Other fields: Neurodegenerative diseases, acute liver failure, brain injury	[54,83,153,154,155]
GM-CSF	PI3K/AKT Ras/MAPKs JAK/STATs	✓ Activation of macrophages; ✓ Differentiation of immune cells; ✓ Cell survival and proliferation.	Reproductive medicine: POI Other fields: Lung injury	[156,157,158]
PDGF (*)	PI3K/AKT JAK/STATs Ras/MAPKs PLC-ϒ	✓ Mitosis and chemotaxis of mesenchymal-origin cells; ✓ Activation of macrophages; ✓ Angiogenesis and blood vessel repair.	Reproductive medicine: IUA Other fields: Chronic diabetic wounds, acute kidney injury, liver fibrosis, lung diseases	[159,160,161,162,163]
TGFβ	Canonical: SMAD Non-canonical: PI3K/AKT Ras/MAPKs	✓ Collagen synthesis; ✓ Angiogenesis; ✓ Cell chemotaxis; ✓ Cell growth; ✓ Inhibition of osteoclast formation and bone resorption; ✓ Promotion of wound healing; ✓ Inhibition of apoptosis; ✓ Remodeling of the ECM.	Reproductive medicine: IUA, POI, breast cancer Other fields: Atopic dermatitis, liver fibrosis, renal fibrosis, lung injury, wounds	[87,164,165,166,167,168]
ILs	PI3K/AKT Ras/MAPKs JAK/STATs	✓ Chemotaxis; ✓ Cell proliferation; ✓ Angiogenesis; ✓ Immune modulation.	Reproductive medicine: IUA, POI, ovarian carcinoma Other fields: Autoimmune encephalitis, neuropathic pain, spondyloarthritis, brain injury, dermatitis	[139,144,169,170,171,172,173]
CKs	PI3K/AKT Ras/MAPKs JAK/STATs PLC-ϒ	✓ Chemotaxis and cell adhesion; ✓ Cell growth; ✓ Cell proliferation; ✓ Release of granules and oxidants.	Reproductive medicine: IUA, POI Other fields: Liver failure, lung injury, brain injury	[94,174,175,176,177,178]

(*) PDGF is secreted by platelets, but it was included in the table because of its key role in acellular therapies. VEGF, vascular endothelial growth factor; NGF, nerve growth factor; EGF, endothelial growth factor; FGF, fibroblast growth factor; HGF, hepatocyte growth factor; G-CSF, granulocyte colony-stimulating factor; GM-CSF, granulocyte–macrophage colony-stimulating factor; PDGF, platelet-derived growth factor; TGFβ, transforming growth factor beta; ILs, interleukins; CKs, chemokines; PI3K, phosphoinositide 3-kinase; MAPKs, mitogen-activated protein kinases; PLC-ϒ, phospholipase C-gamma; JNK, c-Jun N-terminal kinase; ECM, extracellular matrix; IUA, intrauterine adhesions; POI, premature ovarian insufficiency.

## 3. Application of Umbilical Cord Stem Cells and Their Derivatives in the Ovary

### 3.1. Cellular Therapies Based on hUC-MSCs: Current Applications, Administration, and Fertility Restoration

Within the female reproductive tract, the ovary is the organ most commonly treated with hUC cells, especially MSCs. The main reports of the use of hUC-MSCs and UC blood derivatives were included in Table 2. In 2013, Wang et al. [35] pioneered the use of hUC-MSCs to treat POI in mice, restoring ovarian function, serum estrogen levels, adequate follicle development, and notably reducing cell apoptosis.

Similar to how other cell therapies have been infused in rodents [179], hUC-MSCs have been administered via tail vein injection, to foster their natural migration towards the injured site [84,180,181,182,183,184,185,186,187]. This strategy not only improved ovarian morphology, primordial follicle development, and hormone production, but also restored estrous cyclicity [84,181,183,186], implying ovarian performance was recovered. More importantly, systemic treatment with hUC-MSCs restored fertility in all cases [181,185,187]. Furthermore, hUC-MSC therapy provoked ovarian secretion of cytokines such as HGF, VEGF, and insulin growth factor 1 (IGF1), suggesting ovarian regeneration was mediated by paracrine mechanisms, and these factors could improve the ovarian function and delay ovarian senescence [180]. Using metabolomics, Zhao et al. [181] showed that hUC-MSCs activated the phosphoinositide 3-kinase (PI3K) pathway by stimulating synthesis of free amino acids, improved lipid metabolism, and decreased concentration of monosaccharides [181]. Meanwhile, Lu’s group [182] also focused on the protective effect of the hUC-MSCs on the theca-interstitial cells. In vitro, they observed an interesting reduction in autophagy levels in the theca-interstitial cells, induced by the decrease in oxidative stress and regulation of the AMP-activated protein kinase (AMPK)/Mammalian target of rapamycin (mTOR) signaling pathway. Augmented TrkA and NGF following hUC-MSC treatment was also reported by Zheng et al. [84], corroborating the involvement of the NGF/TrkA signaling pathway in ovarian regeneration. Moreover, the efficacy of multiple intravenous injections in a chemotherapy-induced POI mouse model was analyzed by Lv et al. [187]. After introducing cells, the ovarian morphology, follicle count, and fertility improved, regardless of whether mice received a single or triple administration. However, higher levels of serum anti-Müllerian hormone (AMH) and local Ki67 expression suggested that multiple doses of hUC-MSCs have a superior therapeutic effect than a single hUC-MSC bolus.

Alternatively, local intraovarian injections were also reported [37,188,189], and were capable of restoring follicle development and fertility in rodents. Interestingly, Shi et al. [188] analyzed the toxicity associated with injecting increasing doses of hUC-MSCs. The maximum tolerated dose was 10^6^ cells/ovary, with severe toxicity and lethality observed after exceeding this limit. Meanwhile, Pan et al. [189] found that hUC-MSC and amniotic MSC treatment, comparably restored damaged ovarian morphology, functionality, elasticity, and toughness. Finally, intraperitoneal treatment with hUC-MSCs showed similar results in terms of follicle populations, hormone regulation, and fertility restoration [190].

Despite these promising findings, administration routes for hUC-MSCs remain controversial. Three independent groups compared the efficacy of intravenous or local administration for POI models [31,36,191]. In these studies, both routes reduced cell apoptosis, augmented the proportion of growing follicles, and restored the ovarian function (as determined by regulated hormonal levels and recuperated estrous cyclicity). On the other hand, Song’s group [36] did not find any differences, Zhang et al. [191] reasoned there was a better restoration of the ovarian function after intravenous treatment, and Zhu et al. [31] found that local injection was more efficient. In terms of ovarian aging models, Zhang et al. [192] observed the positive effects of hUC-MSCs regardless of the injection route (intravenous or intraovarian), with increased follicle development, restored fertility, and reduced apoptosis of granulosa cells and reactive oxygen species (ROS) production. Finally, in humans, ovarian injection of hUC-MSCs rescued ovarian function, increased follicle development, and ultimately, led to live births [193].

Recently, Lu et al. [194] elegantly studied the impact of hUC-MSCs throughout the ovary and the endometrium. The SCs were introduced via tail injection, two weeks after inducing a POI condition. They reported improved ovarian morphology and folliculogenesis, as well as restored serum hormone levels. Regarding the endometrium, an increased number of glands and enhanced neoangiogenesis were observed, in addition to a noticeable overexpression of Homeobox A10 (HOXA10, an essential regulator of endometrial decidualization [195]), altogether revealing improved morphology and functionality. Furthermore, the type 1/2 T helper (Th1/Th2) cell ratio, and expression of NK cells, significantly decreased in the endometrium, suggesting treatment significantly regulated the ovarian function and endometrial receptivity. Likewise, Aygün et al. [196] loaded hUC-MSCs in hyaluronic acid scaffolds, and transplanted intraperitoneally in an abdominal adhesion rat model, and demonstrated the synergistic effect of the SCs on restoring hormone levels, improving follicle development and endometrial angiogenesis (after reducing the adhesions macroscopically).

While hyaluronic acid is the most popular scaffold used to develop alternative treatments in murine models of ovarian pathologies [197,198], other synthetic scaffolds have been mixed with biological products to enhance their pharmacodynamics [199]. Notably, only Sun’s group [200] has translated the use of collagen scaffolds from mice [201] to humans. Nevertheless, in mice, both hyaluronic acid and collagen scaffolds sustained the effects of the hUC-MSCs, improving ovarian morphology, cell proliferation, angiogenesis, and ultimately, restoring fertility [197,198,201]. Likewise, women treated with the hUC-MSC-collagen complex achieved a complete restoration of fertility, with primordial follicle activation (determined by the expression of phosphorylation of Forkhead box O3a (FOXO3a) and Forkhead box O1 (FOXO 1)), increased proportion of growing follicles, and consequent high serum estradiol concentration [200].

### 3.2. Emerging Alternatives: Acellular Therapies

#### 3.2.1. Extracellular Vesicles

When hUC-MSCs are isolated and applied for regenerative treatments, the bioactive executors of hUC-MSCs effects (including EVs) remain present in the cell secretome [202]. Based on this premise, independent groups have employed the medium collected from hUC-MSC culture as a reparative treatment for POI models [82,197], and found it increased AMH levels and folliculogenesis.

Specifically, several studies have described that hUC-MSCs secrete EVs, such as exosomes and microvesicles, to deliver biomolecules (i.e., lipids, carbohydrates, nucleic acids, and proteins) that facilitate cellular and host cell reprogramming [62,203]. Consistent with the findings of two other independent groups [49,107], Liu et al. [204] reported that administering EVs, derived from the hUC, through the tail vein efficiently improved fertility of mice with POI, by improving ovarian and follicle morphology, balancing hormone levels, returning estrous cyclicity, and reducing apoptosis.

The intraperitoneal transplantation of exosomes also proved beneficial in mice, by returning folliculogenesis and hormones to nearly normal levels [205]. This improvement in reproductive outcomes was associated with the regulation of the Hippo pathway, which is critical for regulating follicle activation and survival, and thus, ovarian function [206]. Furthermore, Ding et al. [48] demonstrated, in a murine model, that exosomal miRNA-17-5p was strongly associated with improvements in ovarian function. Indeed, the inhibition of this miRNA via a knockdown approach in hUC-MSCs diminished the regenerative effects of the exosomes.

#### 3.2.2. Growth Factors

The hUC, and its cells, actively secrete GFs that can potentially be used for regenerative applications, since they stimulate cell proliferation and differentiation [207]. Specifically, the hUC-MSCs are reservoirs that, in response to specific regenerative signals in their microenvironment, release molecules to promote tissue repair. In fact, Wang et al. [208] reported that intraperitoneal treatment with GM-CSFs (which are present in the hUC-MSC secretome and immunomodulate hematopoietic cells [208]) promoted follicle development (as validated through the expression of folliculogenesis-related biomarkers) [158]. Similarly, other GFs, such as HGF (enriched in the hUC-MSC secretome) [198], or EGF (loaded into collagen-based scaffolds as Matrigel^®^) [209] have also been associated with positive fertility outcomes.

#### 3.2.3. Plasma and Platelet-Rich Plasma

In terms of ovarian regeneration, Buigues et al. [61] compared the effect of hUC plasma (rich in many GFs) to that of mobilizing G-CSF treatment and demonstrated that both therapies increased the populations of growing follicles, proliferation, and angiogenesis, in addition to restoring fertility of mice with gonadotoxic damage. Furthermore, Wang et al. [210] observed the ample benefits of hUC-PRP treatment in a murine model of POI, including preserved ovarian morphology, regulated hormonal levels and estrous cyclicity, increased angiogenesis, and reduced apoptosis, with respect to the untreated groups.

**Table 2:** Outcomes of studies applying hUC-MSCs or their derivatives (with or without bioengineered scaffolds) to treat ovarian pathologies.

Treatment	Model	Condition	Administration	Results						Reference
				Ovarian Morphology	Developing Follicles	Serum Hormone Levels	Estrous Cyclicity	Markers of Regeneration and Function	Fertility Outcomes	
*hUC-MSC*	Rat	POI	TV	Improved	Improved	↑ E2, AMH ↓ FSH	NR	↑ HGF, VEGF, IGF1	NR	[180]
*hUC-MSC*	Rat	POI	TV	Improved	Improved	↑ E2, P4, AMH	Restored	↑ Cell proliferation ↓ Apoptosis ↑ AMH, Bcl-2, FSHR ↓ Caspase-3	NR	[186]
*hUC-MSC*	Rat	POI	TV	Improved	Improved	↑ E2, AMH, GnRH ↓ FSH	Restored	↑ Cell proliferation ↓ Apoptosis ↑ NGF, TrkA ↓ FSHR, Caspase-3	NR	[84]
*hUC-MSC*	Rat	POI	TV	Improved	Improved	↑ E2, LH ↓ FSH	NR	↓ Apoptosis	NR	[182]
*hUC-MSC*	Mouse	POI	TV	Improved	Improved	↑ E2 ↓ FSH	Restored	NR	Restored	[181]
*hUC-MSC*	Mouse	POI	TV	Improved	Improved	↑ E2, AMH ↓ FSH	NR	↑ Cell proliferation ↑ FSHR, Inhibin α/β	Restored	[187]
*hUC-MSC (CD146+/−)*	Mouse	POI	TV	Improved	Improved	↑ E2, LH ↓ FSH	NR	↑ Cell proliferation ↑ IL-2, TNFα	Restored	[185]
*hUC-MSC*	Mouse	POI	TV	Improved	Improved	↑ E2, P4 ↓ FSH	NR	↑ IL-4 ↓ IFNγ, NK	NR	[194]
*hUC-MSC*	Mouse	POI	TV	Improved	Improved	↑ E2 ↓ FSH	Restored	NR	NR	[183]
*hUC-MSC*	Mouse	POI	TV	NR	NR	NR	NR	NR	NR	[184]
*hUC-MSC*	Rat	POI	Local	↑	NR	NR	NR	NR	NR	[188]
*hUC-MSC*	Mouse	POI	Local	Improved	Improved	↑ E2, AMH ↓ FSH	NR	NR	Restored	[37]
*hUC-MSC*	Mouse	POI	Local	Improved	NR	↑ E2, LH ↓ FSH	NR	↑ VEGF	Restored	[189]
*hUC-MSC*	Human	POI	Local	Improved	Improved	NR	NR	NR	Restored	[193]
*hUC-MSC*	Rat	POI	IP	NR	Improved	↑ E2, LH ↓ FSH	NR	↓ Fibrosis	Restored	[190]
*hUC-MSC*	Rat	POI	TV vs. local	Improved	Improved	↑ E2, AMH, LH ↓ FSH	Restored	↓ Apoptosis	Restored	[191]
*hUC-MSC*	Rat	POI	TV vs. local	NR	Improved	NR	NR	↓ Apoptosis	NR	[36]
*hUC-MSC*	Rat	POI	TV vs. local	Improved	Improved	↑ E2 ↓ FSH	Restored	NR	Restored	[31]
*hUC-MSC*	Mouse	POI	NR	NR	Improved	↑ E2	NR	↓ Apoptosis	NR	[35]
*hUC-MSC*	Mouse	Aging	TV vs. local	Improved	Improved	↑ E2, P4	Restored	↓ Apoptosis ↓ ROS production	Restored	[192]
*hUC-MSC + collagen*	Mouse	POI	Local	Improved	Improved	↑ AMH, LH ↓ FSH	Restored	↑ Cell proliferation ↑ Angiogenesis	NR	[201]
*hUC-MSC + collagen*	Human	POI	Local	Improved	Improved	↑ E2 ↓ FSH	NR	NR	Restored	[200]
*hUC-MSC + HA*	Mouse	POI	Local	Improved	Improved	NR	NR	↓ Apoptosis	Restored	[197]
*hUC-MSC vs. HGF*	Mouse	POI	Local	NR	Improved	NR	NR	NR	NR	[198]
*hUC-MSC + AF*	Rat	Abdominal adhesions	IP	Improved	Improved	NR	NR	NR	NR	[196]
*hUC-MSC EV*	Mouse	POI	TV	Improved	Improved	↑ E2 ↓ FSH	Restored	↓ Apoptosis	Restored	[204]
*hUC-MSC exosomes*	Mouse	POI	IP	NR	Improved	↑ E2, AMH ↓ FSH	Restored	↑ Cell proliferation	Restored	[205]
*hUC-MSC exosomes*	Mouse	POI	Local	Improved	Improved	↑ E2, AMH ↓ FSH	NR	↑ Cell proliferation ↓ Apoptosis ↓ ROS production	Restored	[48]
*hUC-MSC vesicles*	Mouse	Aging	Local	NR	Improved	↑ E2 ↓ FSH	Restored	↑ Oocyte quality	Restored	[49]
*hUC-MSC microvesicles*	Mouse	POI	Vena caudalis injection	Improved	Improved	↑ E2 ↓ FSH	Restored	↑ Angiogenesis ↑ VEGF, IGF1, Ang, AKT, p-AKT	NR	[107]
*hUC-MSC culture medium*	Mouse	POI	IP	Improved	Improved	↑ AMH	NR	NR	NR	[82]
*hUC-MSC + hUC-PRP*	Rat	POI	Local	Improved	NR	↑ E2, AMH ↓ FSH	Restored	↑ Angiogenesis ↓ Apoptosis	NR	[210]
*G-CSF vs. UC plasma*	Mouse	POI	TV	Improved	Improved	NR	NR	↑ Cell proliferation ↑ Angiogenesis	Restored	[61]
*GM-CSF*	Rat	POI	IP	NR	Improved	NR	NR	↑ CYP17, CD45	NR	[158]
*EGF + Matrigel*	Mouse	POI	Local	NR	Improved	NR	NR	NR	Restored	[209]

The up and down arrows, respectively, indicate an increase and decrease. hUC-MSC, human umbilical cord mesenchymal stem cells; POI, premature ovarian insufficiency; E2, estrogen; AMH, anti-Müllerian hormone; FSH, follicle-stimulating hormone; NR, Non-reported; HGF, hepatocyte growth factor; VEGF, vascular endothelial growth factor; IGF1, insulin growth factor 1; P4, progesterone; Bcl-2, B-cell lymphoma 2; FSHR, follicle-stimulating hormone receptor; GnRH, gonadotropin-releasing hormone; NGF, nerve growth factor; TrkA, tropomyosin receptor kinase A; LH, luteinizing hormone; hUC-PRP, human umbilical cord platelet-rich plasma; CD146, cluster of differentiation 146; IL-2, interleukin 2; TNFα, tumor necrosis factor alpha; IL-4, interleukin 4; IFNγ, interferon gamma; NK, natural killer cells; ROS, reactive oxygen species; HA, hyaluronic acid; HGF, hepatocyte growth factor; AF, amniotic fluid; EVs, extracellular vesicles; AKT, protein kinase B; p-AKT, phosphorylated-protein kinase B; Ang, angiogenin; G-CSF, granulocyte colony-stimulating factor; CYP17, cytochrome P450 17α-hydroxylase/17,20-lyase; CD45, cluster of differentiation 45; GM-CSF, granulocyte–macrophage colony-stimulating factor; EGF, endothelial growth factor; TV, tail vein injection; IP, intraperitoneal injection.

## 4. Application of Umbilical Cord Stem Cells and Their Derivatives in the Endometrium

### 4.1. Cellular Therapies Based on hUC-MSCs: Current Applications, Administration, and Fertility Restoration

There is currently a great discord apropos the gold-standard treatment for endometrial pathologies [211]. Stem cell therapies based on bone marrow-derived MSCs [212] or menstrual blood-derived MSCs [213] have been proposed. However, the beneficial effect of SCs derived from the UC on the endometrium was only reported in rats as of 2017 [39]. Table 3 summarizes the main findings of studies evaluating the potential management of endometrial pathologies by hUC-MSCs and/or hUC blood derivatives.

After endometrial scar formation, Xu et al. [39] found that hUC-MSCs recovered endometrial morphology and restored fertility in a rat model. As previously described, the SCs can be administered locally within the uterine horns, or systemically (in which case they would need to migrate from the injection site), i.e., intraperitoneally [214] or intravenous (to favor cells reaching the endometrium) [40,215]. Zhuang et al. [216] compared the efficacy of a single intravenous hUC-MSC injection with an intravenous injection combined with a local transplant. They found that treatment with hUC-MSCs promoted tissue repair (via overexpression of FGF, VEGF, HGF, and IGF1), and reduced the inflammation (by downregulating TNFα) which was more marked after combined treatment.

A promising hUC-MSC-based therapeutic strategy was evaluated in a recent clinical trial [68]. Patients with difficulty healing after uterine surgery were treated twice, with local injections of hUC-MSCs, via an 18F Foley catheter that was removed 24h later. The participants, who were followed for three and six months after treatment, reported slight improvements in menstrual blood flow, and presented reduced scarring, increased uterine volume and endometrial thickness. Notably, no adverse events were reported.

Bioengineering strategies set the foundation on which to create new biomaterials that facilitate cell adhesion into wounded areas. In this regard, several bioengineering strategies for endometrial therapies, based on hUC-MSCs, have been reported, principally using collagen scaffolds [38,39]. For example, Xin et al. [38] demonstrated that transplanting hUC-MSCs loaded in collagen scaffolds ameliorated uterine morphology, and increased cell proliferation at different time points, via the paracrine mechanisms exerted by VEGFA, TGFβ, and PDGF-BB produced by the cells. Infusions of hUC-MSCs with collagen were also reported to treat AS (characterized by the presence of intrauterine adhesions and fibrosis) and EA (characterized by suboptimal endometrial thickness that hinders embryo implantation) [67,217]. Cao et al. [217] spread the hUC-MSC-collagen complex on the patients’ uterine walls, and found the treatment restored blood flow and produced a functional endometrial within three months. Of note, almost 40% of the patients they treated achieved pregnancy by the end of a 30-month follow-up period, resulting in a total of eight healthy babies. Similarly, Zhang et al. [67] placed the collagen-driven hUC-MSCs in the uterine wall, and the procedure was performed twice. Histological analyses of biopsies confirmed collagen degradation, and hormonal replacement therapy was used to prepare for embryo transfer. Thicker endometria were observed, and ultimately, three patients achieved pregnancy following embryo transfer, and another woman got pregnant naturally.

Other scaffolds were tested in animal models to demonstrate the enhanced capacity of SC repair. Wang et al. [218] acquired human placentas, decellularized amniotic membranes, seeded them with hUC-MSCs, and used them to treat damaged rat uteri. Three estrous cycles later, the endometria had recovered normal thickness and number of glands, overexpressed metalloproteases (i.e., MMP9) and ILs (i.e., IL-4 and IL-10) related to regeneration and had lower expression of pro-inflammatory cytokines (i.e., IL-2, TNFα, and interferon gamma (IFNγ)). However, this treatment was unable to restore fertility. On the other hand, Zhou et al. [219] encapsulated the hUC-MSCs in a synthetic thermosensible hydrogel (Pluronic F-127), which was injected into rats in the right side of uterine horns nine days after they modeled a thin endometrium. This bioengineering strategy enlarged the endometrium and restored its cell proliferation and angiogenesis. Similarly, Wang et al. [220] demonstrated that treatment with hyaluronic acid loaded with hUC-MSCs successfully thickened the endometrium, increasing the number of glands and expression of pro-regenerative factors (i.e., IL-4, IGF1, and EGF), while reducing fibrosis and expression of pro-inflammatory cytokines (i.e., IFNγ), in primates with intrauterine adhesions.

Since micro-RNAs influence processes associated with tissue damage, repair, and regeneration, they may consequently be used to study endometrial regenerative activity [221]. Indeed, Sun et al. [90] found that hUC-MSCs overexpressing miR-455-5p augmented endometrial gland number and reduced fibrosis in a murine model with intrauterine adhesions. The authors argued that these regenerative processes were mediated by the activation of the JAK/STAT3 signaling pathway, which had previously been associated with cardiac subepithelial and hepatic fibrosis [222,223,224]. Alternatively, Zheng et al. [93] transfected hUC-MSCs with miR-330-5p, combined the cells with a fibroin small-intestinal submucosa scaffold, and injected them into the damaged endometrial surface. In this case, superior gland concentration was found to be mediated by the activation of the circPTP4A2-miR-330-5k-PDK2 pathway.

### 4.2. Emerging Alternatives: Acellular Therapies

#### 4.2.1. Extracellular Vesicles

Although EVs are one of the most broadly exploited bioactive agents from the hUC-MSC secretome [225], only one group has employed them for endometrial conditions [226]. Ebrahim et al. [226] assessed the therapeutic efficacy of the EVs secreted by hUC-MSCs, in a rat model of intrauterine adhesions. Endometrial function was considered to be recovered after eight weeks, with the superior number of glands, reduced fibrosis, overexpression of VEGF, and downregulation of inflammatory markers (i.e., TGFβ, TNFα, IL-1, IL-6, and RUNX Family Transcription Factor 2 (RUNX2)). Notably, this regenerative effect was amplified when the treatment was combined with estrogen.

#### 4.2.2. Growth Factors

Thus far, GF reported from hUC secretome therapy has only been tested in combination with bioengineered scaffolds in the reproductive field. Cai’s group [227] combined synthetic gelatin methacrylate (GelMA) with sodium-alginate (a natural polysaccharide) and FGF in microfluidic droplets and applied them in rats, repairing the endometrium. Interestingly, treatment using FGF combined with collagen-binding domains increased angiogenesis and endometrial thickness in rats [228], and restored menstrual blood volume, endometrial thickness, and ability to achieve pregnancy, in addition to reducing scarring, in humans [229]. Similarly, López-Martínez et al. [230] recently assessed the efficacy of a decellularized endometrial extracellular matrix hydrogel (EndoECM) loaded with a cocktail of basic FGF, IGF1, and PDGF, in a murine model of AS/EA [230]. Both groups observed an improvement in endometrial thickness and number of glands, and enhanced neoangiogenesis; however, López-Martínez et al. additionally demonstrated that the EndoECM amplified the effects of the GFs, in terms of cell proliferation and restored fertility.

#### 4.2.3. Plasma and Platelet-Rich Plasma

Plasma contains platelets which are rich in GFs that, when concentrated, can amplify their therapeutic potential in terms of tissue regeneration. Last year, De Miguel-Gómez et al. [127] reported that damaged murine uterine horns treated with UC plasma exhibited higher cell proliferation than those treated with peripheral blood plasma. Their proteomic analyses revealed overexpression of HOXA10, related to endometrial functionality, and proteins involved in angiogenesis, mitotic cell cycle, PI3K/AKT and JAK/STAT signaling pathways. Rodríguez-Eguren et al. [231] characterized thoroughly the composition of hUC-PRP processed after manual and commercial strategies. Then, its regenerative effect alone or driven by the aforementioned EndoECM was evaluated in a mouse model of AS/EA. They demonstrated the combined treatment could promote endometrial thickness, cell proliferation, angiogenesis, and restored endometrial cell function, by overexpression of AKT1, VEGF, and angiogenin. However, fertility restoration was only achieved when hUC-PRP was administered alone.

**Table 3:** Outcomes of studies applying hUC-MSCs or their derivatives (with or without bioengineered scaffolds) to treat endometrial pathologies.

Treatment	Model	Condition	Administration	Results					Reference
				Thickness	Gland Number	Fibrosis	Regeneration and Functionality Markers	Fertility Outcomes	
*hUC-MSC*	Rat	IUA	TV	Improved	Improved	Reduced	↑ Cell proliferation ↑ Angiogenesis ↑ Itga1, Thbs, Laminin, collagen ↓ VWF	Restored	[215]
*hUC-MSC*	Rat	IUA	TV	Improved	Improved	Reduced	↑ Cell proliferation ↑ Angiogenesis ↑ VEGFA, MMP9, CD31 ↓TNFα, IFNγ, IL-2, IL-4, IL-10	Restored	[40]
*hUC-MSC*	Rat	IUA	Local	Improved	Improved	NR	NR	Restored	[232]
*hUC-MSC*	Rat	Thin endometrium	TV + Local	NR	NR	NR	↑ FGF ↓ TNFα	NR	[216]
*hUC-MSC*	Rat	IUA	IP	Improved	Improved	Reduced	↑ Angiogenesis ↓ TGFβ and Smad3	Restored	[214]
*hUC-MSC*	Human	IUA	Local	Improved	NR	Reduced	Restored menstrual cycle	NR	[68]
*hUC-MSC + collagen*	Rat	IUA	Local	Improved	Improved	Reduced	↑ Angiogenesis ↑ MMP9	Restored	[39]
*hUC-MSC + collagen*	Human	IUA	Local	Improved	NR	Reduced	↑ Cell proliferation ↑ PanCK, ERα, PR ↑ VEGFA, TGFβ, PDGF	Restored	[38]
*hUC-MSC + collagen*	Human	Asherman syndrome	Local	Improved	NR	Reduced	↑ Cell proliferation ↑ Angiogenesis ↑ ERα, PR	Restored	[67]
*hUC-MSC + collagen*	Human	IUA	Local	Improved	NR	Reduced	↑ Cell proliferation ↑ Angiogenesis ↑ ERα, PR	Restored	[217]
*hUC-MSC + AMM*	Rat	IUA	Local	Improved	Improved	NR	↑ Keratin, Vimentin, Integrinβ3, IL-4, IL-10, MMP9, KI67 ↓TNFα, IFNγ, IL-2, VEGF	Non Restored	[218]
*hUC-MSC + PF-127*	Rat	Thin endometrium	Local	Improved	Improved	NR	↑ Cell proliferation ↑ Angiogenesis ↑ VEGFA, Nos3	NR	[219]
*hUC-MSC + SF-SIS*	Mouse	IUA	Local	Improved	Improved	Reduced	NR	NR	[93]
*hUC-MSC + HA*	Monkey	IUA	Local	Improved	Improved	Reduced	↑ IL-4, IGF1, EGF ↓ IFNγ	NR	[220]
*hUC-MSC^miR−455−5p^*	Mouse	IUA	NR	NR	Improved	Reduced	↑ JAK2, STAT3 ↓ SOCS3	NR	[90]
*hUC-MSC EVs*	Rat	IUA	IP	NR	Improved	Reduced	↑ VEGF ↓ TGFβ, TNFα, IL-1, IL-6, RUNX2, COL1A1	NR	[226]
*UC plasma*	Mouse	IUA	Local	NR	NR	NR	↑ Cell proliferation ↑ HOXA10, P85, 2aaa, Stat5A, Rhoa	NR	[127]
*hUC-PRP + EndoECM*	Mouse	IUA	Local	Improved	Improved	Reduced	↑ Cell proliferation ↑ Angiogenesis ↑ AKT1, VEGF, angiogenin	Restored	[231]
*PDGF-BB + FGF + IGF1 + EndoECM*	Mouse	IUA	Local	Improved	Improved	Reduced	↑ Cell proliferation ↑ Angiogenesis ↓ Col1A1	Restored	[230]
*FGF + CBD*	Rat	IUA	Local	Improved	NR	Reduced	↑ Angiogenesis	Restored	[228]
*FGF + CBD*	Human	IUA	Local	Improved	NR	Reduced	↑ Cell proliferation ↑ Angiogenesis	Restored	[229]
*FGF + GelMA + Na-alginate scaffold*	Rat	IUA	Local	Improved	Improved	Reduced	↑ Angiogenesis	NR	[227]

The up and down arrows, respectively, indicate an increase and decrease. hUC-MSC, human umbilical cord mesenchymal stem cells; IUA, intrauterine adhesions; Itga1, integrin subunit alpha 1; Thbs, thrombospondin; VWF, Von Willebrand factor; VEGF, vascular endothelial growth factor, VEGFA, vascular endothelial growth factor A; MMP9, metalloprotease 9; CD31, Cluster of differentiation 31; TNFα, tumor necrosis factor alpha; IFNγ, interferon gamma; IL-2, interleukin 2; IL-4, interleukin 4; IL-10, interleukin 10; NR, non-reported; FGF, fibroblast growth factor; TGFβ, transforming growth factor beta; Smad3, SMAD family member 3; PanCK, pan-cytokeratin; ERα, estrogen receptor alpha; PR, progesterone receptor; PDGF, platelet-derived growth factor; AMM, acellular amniotic matrix; PF-127, poly(ethylene oxide)-poly(propylene oxide)-poly(ethylene oxide) 127; Nos3, nitric oxide synthase 3; SF-SIS, silk fibroin small-intestinal submucosa; HA, hyaluronic acid; IGF1, insulin-like growth factor 1; EGF, epidermal growth factor; JAK2, Janus kinase 2; STAT3, signal transducer and activator of transcription 3; SOCS3, suppressor of cytokine signaling 3; miR-455-5p: microRNA 455-5p; EVs: extracellular vesicles; IL-1, interleukin 1; IL-6, interleukin 6; RUNX2, runt-related transcription factor 2; COL1, collagen type 1; HOXA10, homeobox A10; 2aaa, serine/threonine-protein phosphatase 2A; Stat5A, signal transducer and activator of transcription 5A; Rhoa, Ras homolog family member A; CBD, collagen-binding domain; GelMA, gelatin methacrylate; TV, tail vein injection; IP, intraperitoneal injection; EndoECM, decellularized endometrial extracellular matrix hydrogel; UC, umbilical cord; hUC-PRP, human umbilical cord platelet-rich plasma; Na-alginate, sodium alginate.

## 5. Applications of Umbilical Cord Stem Cells and Their Derivatives in Other Female Reproductive Organs

### 5.1. Vagina

Vaginal pelvic organ prolapse is a condition in which one or more organs in the pelvis slip down from their original position and protrude into the vagina [233]. Diverse SC therapies have achieved promising results in preclinical studies, conferring tissue elasticity, muscle regeneration, and reduced inflammatory reactions [234]. Specifically, a rhesus macaque model of pelvic organ prolapse treated with hUC-MSCs showed increased muscular thickness, higher elasticity of the vaginal wall, upregulation of muscular (i.e., COL1A1 and FBN5) and neovascular markers (e.g., VEGF), and downregulation of matrix metalloproteinases (i.e., MMP2/9/13). The innovative strategy of loading hUC-MSCs into synthetic scaffolds was also reported using the same primate model. A scaffold derived from the small intestine submucosa was employed to deliver the hUC-MSCs, which improved angiogenesis, muscular thickness, collagen content, and vaginal wall elasticity, in addition to upregulating muscular markers (i.e., COLI, ACTA, and ELN) and metalloproteases (i.e., MMP1/2), and reorganized the extracellular matrix [235]. Moreover, the use of a collagen I scaffold in a rat model improved the collagen content in the vaginal wall and vaginal elasticity, in addition to upregulating ACTA2, Elastin, FGF, vimentin, COL3A1, TIMP metallopeptidase inhibitor 1 (TIMP1), VEGFA, and TGFβ [236]. Similarly, the use of a synthetic polypropylene scaffold to deliver the hUC-MSCs decreased the inflammatory response, upregulated IL-1, -4, and -10, and downregulated MMP1/9 [237].

We highlight that, so far, only one group has applied hUC-MSCs in humans for vaginal diseases. This particular study was related to stress urinary incontinence (where the urine leaks without bladder contraction), which affects 10–40% of women [238,239]. The hUC-MSCs were injected into the submucosal area of the proximal-urethra, in a two-step procedure, and no adverse events were reported. After measuring urodynamic markers, Lee et al. [240] demonstrated the deficient intrinsic sphincter and mixed stress incontinency were improved by this treatment.

### 5.2. Oviducts

The only application of hUC-MSC within the oviducts was reported in 2019, for chronic salpingitis caused by *Chlamydia trachomatis* [241]. Chronic salpingitis compromises the oviductal structure, leading to distal tube obstruction and hydrosalpinx, which are well-known causes of infertility [242]. Liao et al. [241] pioneered this work by isolating MSCs from UC donors, labelling, and transplanting them into chlamydial-infected mice. Within four weeks, the SC therapy had improved fallopian tube morphology, increased the proliferation index, and reduced apoptosis and inflammation, compared to the non-treated group. Individuals treated with the hUC-MSCs were also capable of achieving pregnancy and live births.

### 5.3. Placenta

Spontaneous abortion is defined as the loss of a pregnancy, without outside intervention, during the first 20 weeks of gestation [242]. The consecutive loss of three or more pregnancies is diagnosed as recurrent pregnancy loss, and may be caused by uterine anatomical defects, chromosomal aberration, or immunological abnormalities [243]. The therapeutic potential of hUC-MSCs in abortion was studied using a rat model generated by Chen et al. [244]. This group induced abortion with bromocriptine, between gestational days 6–8, and transplanted the hUC-MSCs on day 9. This treatment prevented necrosis of the placental decidual cells (usually provoked by abortion), and restored levels of IL-10, -17, and IFNγ to normal, in addition to reducing early pregnancy loss after mating. However, the long-term safety and effectiveness of this treatment merits further investigation.

## 6. Pros and Cons of Using Human Umbilical Cord Stem Cells and Their Derivatives

**Figure 2:**
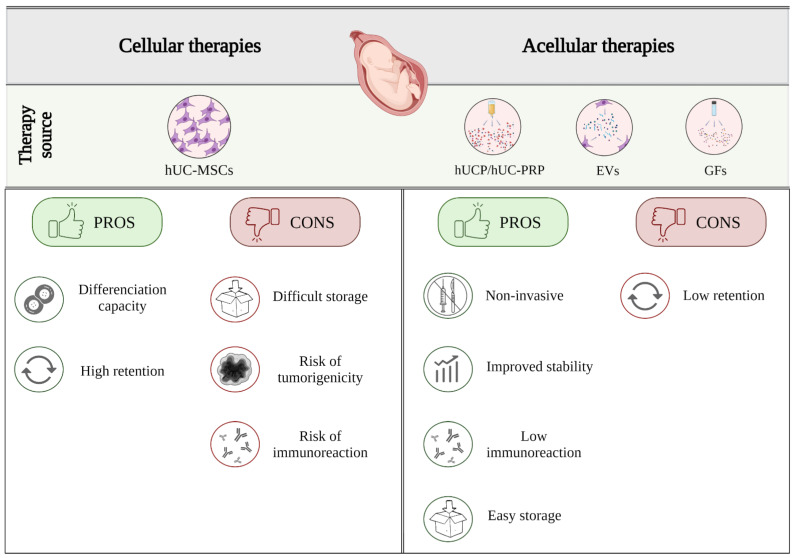
Pros and cons of using cellular and acellular UC therapies in the female reproductive tract. hUC-MSCs: human umbilical cord mesenchymal stem cells; hUCP: human umbilical cord plasma; hUC-PRP: human umbilical cord platelet-rich plasma; EVs: extracellular vesicles; GFs: growth factors.

Despite the application of hUC-MSCs proving to have therapeutic benefits for reproductive diseases in preclinical models, further studies are required to assess their efficacy in the clinical setting and long-term safety (Figure 2). Once they reach their target tissue, the hUC-MSCs can differentiate into resident cells (depending on the signals they receive from the tissue-specific ECM), and thus potentially provide a lifetime supply of cells for the patient, with only a single dose. However, SC therapy has some limitations, including the need to establish a bank of hUC-MSCs, and their tumorigenic potential, which makes clinical translation challenging [245].

Acellular therapies based on the hUC-MSC secretome, or specific hUC blood derivatives, are emerging as promising alternatives in reproductive medicine. Specifically, EVs and hUC-MSCs are postulated to have similar biological effects, but EVs are more advantageous in terms of greater stability, easier storage, diminishing the risk of ectopic tissue formation, possible immune rejection, and tumorigenicity, when compared to cell transplantation (Figure 2) [110]. Similarly, the acellular components of hUC blood, such as hUC plasma, hUC-PRP, and GFs, exhibited lower immunogenicity and tumorigenicity, compared to cell transplantation. Apart from being more economical in the long-term [245], these components are microbiologically safe, and avoid the extra burdens associated with autologous treatments [137]. Furthermore, soluble GFs in the hUCS and hUCP remain stable after extended periods of cryopreservation, facilitating their management [246]. Given these reasons, we expect that the acellular components of human UC blood—particularly PRP—will be key players in the future of regenerative medicine. However, future studies should be conducted to develop strategies that mitigate potential problems with their targeting or retention [225].

Advances in bioengineering offer new methodologies to sustain delivery of cells and paracrine factors, boosting efficiency and maintaining long-term efficacy [199]. Thus far, only collagen-derived scaffolds have been applied in clinical trials, for patients affected by POI [200] and AS [67,217]. However, tissue-specific hydrogel scaffolds that mimic the target microenvironment are quickly emerging as alternatives, since they potentiate the restorative effects [247], as has been demonstrated with murine models of AS [230].

Remarkably, the US Food and Drug Administration (FDA) states the products used in regenerative medicine must be regulated prior to their implementation in the industry and into patients. Specifically, the safety, purity, potency, and identity of the hUC blood or their derivatives, as well as their testing in clinical trials, are mandatory [248].

Due to the lack of studies comparing cellular and acellular therapies, it is difficult to evaluate which of the therapies described herein is superior. Nevertheless, the ongoing clinical trials assessing the application of hUC-PRP in the endometrium [138], and hUC-MSCs in the endometrium [249,250] and ovaries [251,252,253], bring these products one step closer to clinical translation and, ultimately, helping to treat infertility-related conditions. Further information about these ongoing clinical trials is provided in Supplementary Table S1.

Notably, treatments based on hUC blood components are also emerging as alternatives to treat male infertility. Preclinical models have already been used to demonstrate the ability of hUC-MSCs to differentiate into germ cells, in the lumen of the seminiferous tubules [254,255], and their therapeutic effect against the gonadotoxicities of lead [256]. However, research in this field remains experimental, as there have not been any reports of clinical trials evaluating their use to treat male infertility-related conditions [257].

## 7. Conclusions

In conclusion, regenerative medicine targeting female reproduction has generated promising outcomes through the use of hUC-MSCs and blood derivatives, such as hUC-MSCs, hUC-PRP, and EVs, in the treatment of multiple gynecological disorders (i.e., POI, AS, EA, pelvic organ prolapse, or abortions). These treatments enhanced tissue regeneration by upregulating the secretion of GFs and cytokines and through their immunomodulatory actions. Further studies are required to establish a clinical benefit, and to determine whether cellular or acellular therapy is superior.

## Data Availability

Not applicable.

## Supplementary Materials

The supporting information can be downloaded at: https://www.mdpi.com/article/10.3390/ijms232415942/s1.

## References

[1] World Health Organization (WHO) (2018). International Classification of Diseases.

[2] Igboeli P., El Andaloussi A., Sheikh U., Takala H., Elsharoud A., McHugh A., Gavrilova-Jordan L., Levy S., Al-Hendy A. (2020). Intraovarian Injection of Autologous Human Mesenchymal Stem Cells Increases Estrogen Production and Reduces Menopausal Symptoms in Women with Premature Ovarian Failure: Two Case Reports and a Review of the Literature. J. Med. Case Rep..

[3] Zhang Y., Lin X., Dai Y., Hu X., Zhu H., Jiang Y., Zhang S. (2016). Endometrial Stem Cells Repair Injured Endometrium and Induce Angiogenesis via AKT and ERK Pathways. Reproduction.

[4] Lv Q., Wang L., Luo X., Chen X. (2021). Adult Stem Cells in Endometrial Regeneration: Molecular Insights and Clinical Applications. Mol. Reprod. Dev..

[5] Alberts B., Johnson A., Lewis J., Raff M., Roberts K., Walter P., Garland Science (2002). Molecular Biology of the Cell—NCBI Bookshelf.

[6] Naji A., Rouas-Freiss N., Durrbach A., Carosella E.D., Sensébé L., Deschaseaux F. (2013). Concise Review: Combining Human Leukocyte Antigen G and Mesenchymal Stem Cells for Immunosuppressant Biotherapy. Stem Cells.

[7] Squillaro T., Peluso G., Galderisi U. (2016). Clinical Trials with Mesenchymal Stem Cells: An Update. Cell Transplant..

[8] Galipeau J., Sensébé L. (2018). Mesenchymal Stromal Cells: Clinical Challenges and Therapeutic Opportunities. Cell Stem Cell.

[9] Trounson A., McDonald C. (2015). Stem Cell Therapies in Clinical Trials: Progress and Challenges. Cell Stem Cell.

[10] Fazeli Z., Abedindo A., Omrani M.D., Ghaderian S.M.H. (2018). Mesenchymal Stem Cells (MSCs) Therapy for Recovery of Fertility: A Systematic Review. Stem Cell Rev. Rep..

[11] Zhao Y.X., Chen S.R., Su P.P., Huang F.H., Shi Y.C., Shi Q.Y., Lin S. (2019). Using Mesenchymal Stem Cells to Treat Female Infertility: An Update on Female Reproductive Diseases. Stem Cells Int..

[12] Esfandyari S., Chugh R.M., Park H.S., Hobeika E., Ulin M., Al-Hendy A. (2020). Mesenchymal Stem Cells as a Bio Organ for Treatment of Female Infertility. Cells.

[13] Rungsiwiwut R., Virutamasen P., Pruksananonda K. (2020). Mesenchymal Stem Cells for Restoring Endometrial Function: An Infertility Perspective. Reprod. Med. Biol..

[14] Chang Z., Zhu H., Zhou X., Zhang Y., Jiang B., Li S., Chen L., Pan X., Feng X.L. (2021). Mesenchymal Stem Cells in Preclinical Infertility Cytotherapy: A Retrospective Review. Stem Cells Int..

[15] Lorzadeh N., Kazemirad N. (2018). Application of Stem Cells to Infertility Treatment with Emphasis on Mesenchymal Stem Cells and Ovarian Stem Cells. Am. J. Perinatol..

[16] Mohamed S.A., Shalaby S.M., Abdelaziz M., Brakta S., Hill W.D., Ismail N., Al-Hendy A. (2018). Human Mesenchymal Stem Cells Partially Reverse Infertility in Chemotherapy-Induced Ovarian Failure. Reprod. Sci..

[17] Ullah I., Subbarao R.B., Rho G.J. (2015). Human Mesenchymal Stem Cells—Current Trends and Future Prospective. Biosci. Rep..

[18] Spitzhorn L.S., Megges M., Wruck W., Rahman M.S., Otte J., Degistirici Ö., Meisel R., Sorg R.V., Oreffo R.O.C., Adjaye J. (2019). Human IPSC-Derived MSCs (IMSCs) from Aged Individuals Acquire a Rejuvenation Signature. Stem Cell Res. Ther..

[19] Fong C.Y., Chak L.L., Biswas A., Tan J.H., Gauthaman K., Chan W.K., Bongso A. (2011). Human Wharton’s Jelly Stem Cells Have Unique Transcriptome Profiles Compared to Human Embryonic Stem Cells and Other Mesenchymal Stem Cells. Stem Cell Rev. Rep..

[20] Nagamura-Inoue T., He H. (2014). Umbilical Cord-Derived Mesenchymal Stem Cells: Their Advantages and Potential Clinical Utility. World J. Stem Cells.

[21] Zhang C. (2020). The Roles of Different Stem Cells in Premature Ovarian Failure. Curr. Stem Cell Res. Ther..

[22] Yu Y.B., Song Y., Chen Y., Zhang F., Qi F.Z. (2018). Differentiation of Umbilical Cord Mesenchymal Stem Cells into Hepatocytes in Comparison with Bone Marrow Mesenchymal Stem Cells. Mol. Med. Rep..

[23] Qiu Y., Yun M.M., Han X., Zhao R., Zhou E., Yun S. (2014). Human Umbilical Cord Mesenchymal Stromal Cells Suppress MHC Class II Expression on Rat Vascular Endothelium and Prolong Survival Time of Cardiac Allograft. Int. J. Clin. Exp. Med..

[24] Alanazi A., Alassiri M., Jawdat D., Almalik Y. (2022). Mesenchymal Stem Cell Therapy: A Review of Clinical Trials for Multiple Sclerosis. Regen. Ther..

[25] Jin J.-L., Liu Z., Lu Z.-J., Guan D.-N., Wang C., Chen Z.-B., Zhang J., Zhang W.-Y., Wu J.-Y., Xu Y. (2013). Safety and Efficacy of Umbilical Cord Mesenchymal Stem Cell Therapy in Hereditary Spinocerebellar Ataxia. Curr. Neurovasc. Res..

[26] Guan L.X., Guan H., Li H.B., Ren C.A., Liu L., Chu J.J., Dai L.J. (2015). Therapeutic Efficacy of Umbilical Cord-Derived Mesenchymal Stem Cells in Patients with Type 2 Diabetes. Exp. Ther. Med..

[27] Cheng H., Liu X., Hua R., Dai G., Wang X., Gao J., An Y. (2014). Clinical Observation of Umbilical Cord Mesenchymal Stem Cell Transplantation in Treatment for Sequelae of Thoracolumbar Spinal Cord Injury. J. Transl. Med..

[28] Xue H.L., Zeng W.Z., Wu X.L., Jiang M.D., Zheng S.M., Zhang Y., Li H.Y. (2015). Clinical Therapeutic Effects of Human Umbilical Cord–Derived Mesenchymal Stem Cells Transplantation in the Treatment of End-Stage Liver Disease. Transplant. Proc..

[29] Ding D.C., Chang Y.H., Shyu W.C., Lin S.Z. (2015). Human Umbilical Cord Mesenchymal Stem Cells: A New Era for Stem Cell Therapy. Cell Transplant..

[30] Cao M., Chan R.W.S., Yeung W.S.B. (2015). Label-Retaining Stromal Cells in Mouse Endometrium Awaken for Expansion and Repair after Parturition. Stem Cells Dev..

[31] Zhu S.F., Hu H.B., Xu H.Y., Fu X.F., Peng D.X., Su W.Y., He Y.L. (2015). Human Umbilical Cord Mesenchymal Stem Cell Transplantation Restores Damaged Ovaries. J. Cell Mol. Med..

[32] Yang X., Zhang M., Zhang Y., Li W., Yang B. (2011). Mesenchymal Stem Cells Derived from Wharton Jelly of the Human Umbilical Cord Ameliorate Damage to Human Endometrial Stromal Cells. Fertil. Steril..

[33] Fan D., Wu S., Ye S., Wang W., Guo X., Liu Z. (2017). Umbilical Cord Mesenchyme Stem Cell Local Intramuscular Injection for Treatment of Uterine Niche: Protocol for a Prospective, Randomized, Double-Blinded, Placebo-Controlled Clinical Trial. Medicine.

[34] Shi Q., Gao J., Jiang Y., Sun B., Lu W., Su M., Xu Y., Yang X., Zhang Y. (2017). Differentiation of Human Umbilical Cord Wharton’s Jelly-Derived Mesenchymal Stem Cells into Endometrial Cells. Stem Cell Res. Ther..

[35] Wang S., Yu L., Sun M., Mu S., Wang C., Wang D., Yao Y. (2013). The Therapeutic Potential of Umbilical Cord Mesenchymal Stem Cells in Mice Premature Ovarian Failure. Biomed Res. Int..

[36] Li H., Song D., Zhong Y., Qian C., Zou Q., Ou J., Shi Y., Gao L., Wang G., Liu Z. (2016). Human Umbilical Cord Mesenchymal Stem Cells Therapy in Cyclophosphamide-Induced Premature Ovarian Failure Rat Model. Biomed Res. Int..

[37] Mohamed S.A., Shalaby S., Brakta S., Elam L., Elsharoud A., Al-Hendy A. (2019). Umbilical Cord Blood Mesenchymal Stem Cells as an Infertility Treatment for Chemotherapy Induced Premature Ovarian Insufficiency. Biomedicines.

[38] Xin L., Lin X., Pan Y., Zheng X., Shi L., Zhang Y., Ma L., Gao C., Zhang S. (2019). A Collagen Scaffold Loaded with Human Umbilical Cord-Derived Mesenchymal Stem Cells Facilitates Endometrial Regeneration and Restores Fertility. Acta Biomater..

[39] Xu L., Ding L., Wang L., Cao Y., Zhu H., Lu J., Li X., Song T., Hu Y., Dai J. (2017). Umbilical Cord-Derived Mesenchymal Stem Cells on Scaffolds Facilitate Collagen Degradation via Upregulation of MMP-9 in Rat Uterine Scars. Stem Cell Res. Ther..

[40] Zhang L., Li Y., Guan C.Y., Tian S., Lv X.D., Li J.H., Ma X., Xia H.F. (2018). Therapeutic Effect of Human Umbilical Cord-Derived Mesenchymal Stem Cells on Injured Rat Endometrium during Its Chronic Phase. Stem Cell Res. Ther..

[41] Azizi R., Aghebati-Maleki L., Nouri M., Marofi F., Negargar S., Yousefi M. (2018). Stem Cell Therapy in Asherman Syndrome and Thin Endometrium: Stem Cell- Based Therapy. Biomed Pharmacother..

[42] Benor A., Gay S., DeCherney A. (2020). An Update on Stem Cell Therapy for Asherman Syndrome. J. Assist. Reprod. Genet..

[43] Mei Q., Mou H., Liu X., Xiang W. (2021). Therapeutic Potential of HUMSCs in Female Reproductive Aging. Front. Cell Dev. Biol..

[44] György B., Szabó T.G., Pásztói M., Pál Z., Misják P., Aradi B., László V., Pállinger É., Pap E., Kittel Á. (2011). Membrane Vesicles, Current State-of-the-Art: Emerging Role of Extracellular Vesicles. Cell Mol. Life Sci..

[45] Bidarimath M., Khalaj K., Kridli R.T., Kan F.W.K., Koti M., Tayade C. (2017). Extracellular Vesicle Mediated Intercellular Communication at the Porcine Maternal-Fetal Interface: A New Paradigm for Conceptus-Endometrial Cross-Talk. Sci. Rep..

[46] Moghadasi S., Elveny M., Rahman H.S., Suksatan W., Jalil A.T., Abdelbasset W.K., Yumashev A.V., Shariatzadeh S., Motavalli R., Behzad F. (2021). A Paradigm Shift in Cell-Free Approach: The Emerging Role of MSCs-Derived Exosomes in Regenerative Medicine. J. Transl. Med..

[47] Sun L., Li D., Song K., Wei J., Yao S., Li Z., Su X., Ju X., Chao L., Deng X. (2017). Exosomes Derived from Human Umbilical Cord Mesenchymal Stem Cells Protect against Cisplatin-Induced Ovarian Granulosa Cell Stress and Apoptosis in Vitro. Sci. Rep..

[48] Ding C., Zhu L., Shen H., Lu J., Zou Q., Huang C., Li H., Huang B. (2020). Exosomal MiRNA-17-5p Derived from Human Umbilical Cord Mesenchymal Stem Cells Improves Ovarian Function in Premature Ovarian Insufficiency by Regulating SIRT7. Stem Cells.

[49] Yang W., Zhang J., Xu B., He Y., Liu W., Li J., Zhang S., Lin X., Su D., Wu T. (2020). HucMSC-Derived Exosomes Mitigate the Age-Related Retardation of Fertility in Female Mice. Mol. Ther..

[50] Roura S., Farré J., Hove-Madsen L., Prat-Vidal C., Soler-Botija C., Gálvez-Montón C., Vilalta M., Bayes-Genis A. (2010). Exposure to Cardiomyogenic Stimuli Fails to Transdifferentiate Human Umbilical Cord Blood-Derived Mesenchymal Stem Cells. Basic Res. Cardiol..

[51] Roura S., Bagó J.R., Soler-Botija C., Pujal J.M., Gálvez-Montón C., Prat-Vidal C., Llucià-Valldeperas A., Blanco J., Bayes-Genis A. (2012). Human Umbilical Cord Blood-Derived Mesenchymal Stem Cells Promote Vascular Growth in Vivo. PLoS ONE.

[52] Zhang L., Xu W.H., Fu X.H., Huang Q.X., Guo X.Y., Zhang L., Li S.S., Zhu J., Shu J. (2018). Therapeutic Role of Granulocyte Colony-Stimulating Factor (G-CSF) for Infertile Women under in Vitro Fertilization and Embryo Transfer (IVF-ET) Treatment: A Meta-Analysis. Arch. Gynecol. Obstet..

[53] Castellano J.M., Mosher K.I., Abbey R.J., McBride A.A., James M.L., Berdnik D., Shen J.C., Zou B., Xie X.S., Tingle M. (2017). Human Umbilical Cord Plasma Proteins Revitalize Hippocampal Function in Aged Mice. Nature.

[54] Ehrhart J., Sanberg P.R., Garbuzova-Davis S. (2018). Plasma Derived from Human Umbilical Cord Blood: Potential Cell-Additive or Cell-Substitute Therapeutic for Neurodegenerative Diseases. J. Cell Mol. Med..

[55] Murphy M.B., Blashki D., Buchanan R.M., Yazdi I.K., Ferrari M., Simmons P.J., Tasciotti E. (2012). Adult and Umbilical Cord Blood-Derived Platelet-Rich Plasma for Mesenchymal Stem Cell Proliferation, Chemotaxis, and Cryo-Preservation. Biomaterials.

[56] Lehallier B., Gate D., Schaum N., Nanasi T., Lee S.E., Yousef H., Moran Losada P., Berdnik D., Keller A., Verghese J. (2019). Undulating Changes in Human Plasma Proteome Profiles across the Lifespan. Nat. Med..

[57] Gelmetti A., Greppi N., Guez S., Grassi F., Rebulla P., Tadini G. (2018). Cord Blood Platelet Gel for the Treatment of Inherited Epidermolysis Bullosa. Transfus. Apher. Sci..

[58] Tadini G., Pezzani L., Ghirardello S., Rebulla P., Esposito S., Mosca F. (2015). Case Report: Cord Blood Platelet Gel Treatment of Dystrophic Recessive Epidermolysis Bullosa. BMJ Case Rep..

[59] Piccin A., Rebulla P., Pupella S., Tagnin M., Marano G., Di Pierro A.M., Santodirocco M., Di Mauro L., Beqiri L., Kob M. (2017). Impressive Tissue Regeneration of Severe Oral Mucositis Post Stem Cell Transplantation Using Cord Blood Platelet Gel. Transfusion.

[60] Volpe P., Marcuccio D., Stilo G., Alberti A., Foti G., Volpe A., Princi D., Surace R., Pucci G., Massara M. (2017). Efficacy of Cord Blood Platelet Gel Application for Enhancing Diabetic Foot Ulcer Healing after Lower Limb Revascularization. Semin. Vasc. Surg..

[61] Buigues A., Marchante M., de Miguel-Gómez L., Martinez J., Cervelló I., Pellicer A., Herraiz S. (2021). Stem Cell-Secreted Factor Therapy Regenerates the Ovarian Niche and Rescues Follicles. Am. J. Obstet. Gynecol..

[62] Jiao Y., Li X.Y., Liu J. (2019). A New Approach to Cerebral Palsy Treatment: Discussion of the Effective Components of Umbilical Cord Blood and Its Mechanisms of. Cell Transplant..

[63] He X., Wang Q., Zhao Y., Zhang H., Wang B., Pan J., Li J., Yu H., Wang L., Dai J. (2020). Effect of Intramyocardial Grafting Collagen Scaffold with Mesenchymal Stromal Cells in Patients with Chronic Ischemic Heart Disease: A Randomized Clinical Trial. JAMA Netw. Open.

[64] Bartolucci J., Verdugo F.J., González P.L., Larrea R.E., Abarzua E., Goset C., Rojo P., Palma I., Lamich R., Pedreros P.A. (2017). Safety and Efficacy of the Intravenous Infusion of Umbilical Cord Mesenchymal Stem Cells in Patients with Heart Failure: A Phase 1/2 Randomized Controlled Trial (RIMECARD Trial [Randomized Clinical Trial of Intravenous Infusion Umbilical Cord Mesenchymal. Circ. Res..

[65] Özmert E., Arslan U. (2020). Management of Retinitis Pigmentosa by Wharton’s Jelly-Derived Mesenchymal Stem Cells: Prospective Analysis of 1-Year Results. Stem Cell Res. Ther..

[66] Zang L., Li Y., Hao H., Liu J., Cheng Y., Li B., Yin Y., Zhang Q., Gao F., Wang H. (2022). Efficacy and Safety of Umbilical Cord-Derived Mesenchymal Stem Cells in Chinese Adults with Type 2 Diabetes: A Single-Center, Double-Blinded, Randomized, Placebo-Controlled Phase II Trial. Stem Cell Res. Ther..

[67] Zhang Y., Shi L., Lin X., Zhou F., Xin L., Xu W., Yu H., Li J., Pan M., Pan Y. (2021). Unresponsive Thin Endometrium Caused by Asherman Syndrome Treated with Umbilical Cord Mesenchymal Stem Cells on Collagen Scaffolds: A Pilot Study. Stem Cell Res. Ther..

[68] Huang J., Li Q., Yuan X., Liu Q., Zhang W., Li P. (2022). Intrauterine Infusion of Clinically Graded Human Umbilical Cord-Derived Mesenchymal Stem Cells for the Treatment of Poor Healing after Uterine Injury: A Phase I Clinical Trial. Stem Cell Res. Ther..

[69] Benvenuto F., Voci A., Carminati E., Gualandi F., Mancardi G., Uccelli A., Vergani L. (2015). Human Mesenchymal Stem Cells Target Adhesion Molecules and Receptors Involved in T Cell Extravasation. Stem Cell Res. Ther..

[70] Jin W., Liang X., Brooks A., Futrega K., Liu X., Doran M.R., Simpson M.J., Roberts M.S., Wang H. (2018). Modelling of the SDF-1/CXCR4 Regulated in Vivo Homing of Therapeutic Mesenchymal Stem/Stromal Cells in Mice. PeerJ.

[71] Combadiere C., Ahuja S.K., van Damme J., Tiffany H.L., Gao J.L., Murphy P.M. (1995). Monocyte Chemoattractant Protein-3 Is a Functional Ligand for CC Chemokine Receptors 1 and 2B. J. Biol. Chem..

[72] Krafts K.P. (2010). Tissue Repair: The Hidden Drama. Organogenesis.

[73] Zhao M., Rotgans B., Wang T., Cummins S.F. (2016). REGene: A Literature-Based Knowledgebase of Animal Regeneration That Bridge Tissue Regeneration and Cancer. Sci. Rep..

[74] Andrzejewska A., Lukomska B., Janowski M. (2019). Concise Review: Mesenchymal Stem Cells: From Roots to Boost. Stem Cells.

[75] Kim Y.J., Broxmeyer H.E. (2011). Immune Regulatory Cells in Umbilical Cord Blood and Their Potential Roles in Transplantation Tolerance. Crit. Rev. Oncol. Hematol..

[76] Chen L., Xiang E., Li C., Han B., Zhang Q., Rao W., Xiao C., Wu D. (2020). Umbilical Cord-Derived Mesenchymal Stem Cells Ameliorate Nephrocyte Injury and Proteinuria in a Diabetic Nephropathy Rat Model. J. Diabetes Res..

[77] Li H., Rong P., Ma X., Nie W., Chen Y., Zhang J., Dong Q., Yang M., Wang W. (2020). Mouse Umbilical Cord Mesenchymal Stem Cell Paracrine Alleviates Renal Fibrosis in Diabetic Nephropathy by Reducing Myofibroblast Transdifferentiation and Cell Proliferation and Upregulating MMPs in Mesangial Cells. J. Diabetes Res..

[78] Xiang E., Han B., Zhang Q., Rao W., Wang Z., Chang C., Zhang Y., Tu C., Li C., Wu D. (2020). Human Umbilical Cord-Derived Mesenchymal Stem Cells Prevent the Progression of Early Diabetic Nephropathy through Inhibiting Inflammation and Fibrosis. Stem Cell Res. Ther..

[79] Barretto T.A., Park E., Telliyan T., Liu E., Gallagher D., Librach C., Baker A. (2021). Vascular Dysfunction after Modeled Traumatic Brain Injury Is Preserved with Administration of Umbilical Cord Derived Mesenchymal Stromal Cells and Is Associated with Modulation of the Angiogenic Response. J. Neurotrauma.

[80] Mukai T., di Martino E., Tsuji S., Blomgren K., Nagamura-Inoue T., Ådén U. (2021). Umbilical Cord-Derived Mesenchymal Stromal Cells Immunomodulate and Restore Actin Dynamics and Phagocytosis of LPS-Activated Microglia via PI3K/Akt/Rho GTPase Pathway. Cell Death Discov..

[81] Song Y., Wang B., Zhu X., Hu J., Sun J., Xuan J., Ge Z. (2021). Human Umbilical Cord Blood–Derived MSCs Exosome Attenuate Myocardial Injury by Inhibiting Ferroptosis in Acute Myocardial Infarction Mice. Cell Biol. Toxicol..

[82] Hong L., Yan L., Xin Z., Hao J., Liu W., Wang S., Liao S., Wang H., Yang X. (2020). Protective Effects of Human Umbilical Cord Mesenchymal Stem Cell-Derived Conditioned Medium on Ovarian Damage. J. Mol. Cell Biol..

[83] Shareghi-oskoue O., Aghebati-Maleki L., Yousefi M. (2021). Transplantation of Human Umbilical Cord Mesenchymal Stem Cells to Treat Premature Ovarian Failure. Stem Cell Res. Ther..

[84] Zheng Q., Fu X., Jiang J., Zhang N., Zou L., Wang W., Ding M., Chen H. (2019). Umbilical Cord Mesenchymal Stem Cell Transplantation Prevents Chemotherapy-Induced Ovarian Failure via the NGF/TrkA Pathway in Rats. Biomed Res. Int..

[85] Elfayomy A.K., Almasry S.M., El-Tarhouny S.A., Eldomiaty M.A. (2016). Human Umbilical Cord Blood-Mesenchymal Stem Cells Transplantation Renovates the Ovarian Surface Epithelium in a Rat Model of Premature Ovarian Failure: Possible Direct and Indirect Effects. Tissue Cell.

[86] Wang B., Jia H., Zhang B., Wang J., Ji C., Zhu X., Yan Y., Yin L., Yu J., Qian H. (2017). Pre-Incubation with HucMSC-Exosomes Prevents Cisplatin-Induced Nephrotoxicity by Activating Autophagy. Stem Cell Res. Ther..

[87] Li T., Yan Y., Wang B., Qian H., Zhang X., Shen L., Wang M., Zhou Y., Zhu W., Li W. (2013). Exosomes Derived from Human Umbilical Cord Mesenchymal Stem Cells Alleviate Liver Fibrosis. Stem Cells Dev..

[88] Xin L., Lin X., Zhou F., Li C., Wang X., Yu H., Pan Y., Fei H., Ma L., Zhang S. (2020). A Scaffold Laden with Mesenchymal Stem Cell-Derived Exosomes for Promoting Endometrium Regeneration and Fertility Restoration through Macrophage Immunomodulation. Acta Biomater..

[89] Zhao Y., Pan S., Wu X. (2022). Human Umbilical Cord Mesenchymal Stem Cell-Derived Exosomes Inhibit Ovarian Granulosa Cells Inflammatory Response through Inhibition of NF-ΚB Signaling in Polycystic Ovary Syndrome. J. Reprod. Immunol..

[90] Sun D., Jiang Z., Chen Y., Shang D., Miao P., Gao J. (2021). MiR-455-5p Upregulation in Umbilical Cord Mesenchymal Stem Cells Attenuates Endometrial Injury and Promotes Repair of Damaged Endometrium via Janus Kinase/Signal Transducer and Activator of Transcription 3 Signaling. Bioengineered.

[91] Cai M.H., Chen X.Y., Fu L.Q., Du W.L., Yang X., Mou X.Z., Hu P.Y. (2021). Design and Development of Hybrid Hydrogels for Biomedical Applications: Recent Trends in Anticancer Drug Delivery and Tissue Engineering. Front. Bioeng. Biotechnol..

[92] Cheng T., Ding S., Liu S., Li Y., Sun L. (2021). Human Umbilical Cord-Derived Mesenchymal Stem Cell Therapy Ameliorates Lupus through Increasing CD4+ T Cell Senescence via MiR-199a-5p/Sirt1/P53 Axis. Theranostics.

[93] Zheng Y., Li L., Bi X., Xue R. (2022). CircPTP4A2-MiR-330-5p-PDK2 Signaling Facilitates In Vivo Survival of HuMSCs on SF-SIS Scaffolds and Improves the Repair of Damaged Endometrium. Oxid. Med. Cell Longev..

[94] Chen X.Y., Chen Y.Y., Lin W., Chen C.H., Wen Y.C., Hsiao T.C., Chou H.C., Chung K.F., Chuang H.C. (2021). Therapeutic Potential of Human Umbilical Cord-Derived Mesenchymal Stem Cells in Recovering from Murine Pulmonary Emphysema Under Cigarette Smoke Exposure. Front. Med..

[95] El Omar R., Beroud J., Stoltz J.F., Menu P., Velot E., Decot V. (2014). Umbilical Cord Mesenchymal Stem Cells: The New Gold Standard for Mesenchymal Stem Cell-Based Therapies?. Tissue Eng. Part B Rev..

[96] Che N., Li X., Zhou S., Liu R., Shi D., Lu L., Sun L. (2012). Umbilical Cord Mesenchymal Stem Cells Suppress B-Cell Proliferation and Differentiation. Cell Immunol..

[97] Chatterjee D., Marquardt N., Tufa D., Beauclair G., Low H., Hatlapatka T., Hass R., Kasper C., von Kaisenberg C., Schmidt R. (2014). Role of Gamma-Secretase in Human Umbilical-Cord Derived Mesenchymal Stem Cell Mediated Suppression of NK Cell Cytotoxicity. Cell Commun. Signal..

[98] Jerkic M., Gagnon S., Rabani R., Ward-Able T., Masterson C., Otulakowski G., Curley G.F., Marshall J., Kavanagh B.P., Laffey J.G. (2020). Human Umbilical Cord Mesenchymal Stromal Cells Attenuate Systemic Sepsis in Part by Enhancing Peritoneal Macrophage Bacterial Killing via Heme Oxygenase-1 Induction in Rats. Anesthesiology.

[99] Yin N., Wu C., Qiu J., Zhang Y., Bo L., Xu Y., Shi M., Zhu S., Yang G., Mao C. (2020). Protective Properties of Heme Oxygenase-1 Expressed in Umbilical Cord Mesenchymal Stem Cells Help Restore the Ovarian Function of Premature Ovarian Failure Mice through Activating the JNK/Bcl-2 Signal Pathway-Regulated Autophagy and Upregulating the Circulating of CD8+CD28− T cells. Stem Cell Res. Ther..

[100] Kim J., Kim B., Kim S., Lee Y.I., Kim J., Lee J.H. (2020). The Effect of Human Umbilical Cord Blood-Derived Mesenchymal Stem Cell Media Containing Serum on Recovery after Laser Treatment: A Double-Blinded, Randomized, Split-Face Controlled Study. J. Cosmet. Dermatol..

[101] Hoss E., Kollipara R., Alhaddad M., Boen M., Goldman M.P. (2020). Red Deer Umbilical Cord-Derived Stem Cell Conditioned Media Combined with Ablative Resurfacing of the Face. J. Drugs Dermatol..

[102] Alhaddad M., Boen M., Wu D.C., Goldman M.P. (2019). Red Deer Umbilical Cord Lining Mesenchymal Stem Cell Extract Cream for Rejuvenation of the Face. J. Drugs Dermatol..

[103] Shin S., Shin J.U., Lee Y., Kwon T.G., Lee J.H. (2017). The Effects of a Multigrowth Factor-Containing Cream on Recovery after Laser Treatment: A Double-Blinded, Randomized, Split-Face Controlled Study. J. Cosmet. Dermatol..

[104] Pawitan J.A. (2014). Prospect of Stem Cell Conditioned Medium in Regenerative Medicine. Biomed Res. Int..

[105] Phinney D.G., Pittenger M.F. (2017). Concise Review: MSC-Derived Exosomes for Cell-Free Therapy. Stem Cells.

[106] Wang L., Gu Z., Zhao X., Yang N., Wang F., Deng A., Zhao S., Luo L., Wei H., Guan L. (2016). Extracellular Vesicles Released from Human Umbilical Cord-Derived Mesenchymal Stromal Cells Prevent Life-Threatening Acute Graft-Versus-Host Disease in a Mouse Model of Allogeneic Hematopoietic Stem Cell Transplantation. Stem Cells Dev..

[107] Yang Z., Du X., Wang C., Zhang J., Liu C., Li Y., Jiang H. (2019). Therapeutic Effects of Human Umbilical Cord Mesenchymal Stem Cell-Derived Microvesicles on Premature Ovarian Insufficiency in Mice. Stem Cell Res. Ther..

[108] Zhou Y., Xu H., Xu W., Wang B., Wu H., Tao Y., Zhang B., Wang M., Mao F., Yan Y. (2013). Exosomes Released by Human Umbilical Cord Mesenchymal Stem Cells Protect against Cisplatin-Induced Renal Oxidative Stress and Apoptosis in Vivo and in Vitro. Stem Cell Res. Ther..

[109] Baharlooi H., Nouraei Z., Azimi M., Moghadasi A.N., Tavassolifar M.J., Moradi B., Sahraian M.A., Izad M. (2021). Umbilical Cord Mesenchymal Stem Cells as Well as Their Released Exosomes Suppress Proliferation of Activated PBMCs in Multiple Sclerosis. Scand J. Immunol..

[110] Yang J., Chen Z., Pan D., Li H., Shen J. (2020). Umbilical Cord-Derived Mesenchymal Stem Cell-Derived Exosomes Combined Pluronic F127 Hydrogel Promote Chronic Diabetic Wound Healing and Complete Skin Regeneration. Int. J. Nanomed..

[111] Zhang B., Wang M., Gong A., Zhang X., Wu X., Zhu Y., Shi H., Wu L., Zhu W., Qian H. (2015). HucMSc-Exosome Mediated-Wnt4 Signaling Is Required for Cutaneous Wound Healing. Stem Cells.

[112] Nakamura Y., Miyaki S., Ishitobi H., Matsuyama S., Nakasa T., Kamei N., Akimoto T., Higashi Y., Ochi M. (2015). Mesenchymal-Stem-Cell-Derived Exosomes Accelerate Skeletal Muscle Regeneration. FEBS Lett.

[113] Burrello J., Monticone S., Gai C., Gomez Y., Kholia S., Camussi G. (2016). Stem Cell-1. Burrello, J. et al. Stem Cell-Derived Extracellular Vesicles and Immune-Modulation. Front. Cell Dev. Biol..

[114] Maharajan N., Cho G.W., Choi J.H., Jang C.H. (2021). Regenerative Therapy Using Umbilical Cord Serum. In Vivo.

[115] Romanov Y.A., Vtorushina V.V., Dugina T.N., Romanov A.Y., Petrova N.V. (2019). Human Umbilical Cord Blood Serum/Plasma: Cytokine Profile and Prospective Application in Regenerative Medicine. Bull Exp Biol Med..

[116] Hassan G., Kasem I., Soukkarieh C., Aljamali M. (2017). A Simple Method to Isolate and Expand Human Umbilical Cord Derived Mesenchymal Stem Cells: Using Explant Method and Umbilical Cord Blood Serum. Int. J. Stem Cells.

[117] Ang L.P.K., Do T.P., Thein Z.M., Reza H.M., Tan X.W., Yap C., Tan D.T.H., Beuerman R.W. (2011). Ex Vivo Expansion of Conjunctival and Limbal Epithelial Cells Using Cord Blood Serum-Supplemented Culture Medium. Invest. Ophthalmol. Vis. Sci..

[118] Chakraborty A., Dutta J., Das S., Datta H. (2013). Effect of Cord Blood Serum on Ex Vivo Human Limbal Epithelial Cell Culture. J. Ocul. Biol. Dis. Informatics.

[119] Zhang Z.G., Zhao J.H., Wei Z.L., Cong L., Zhou P., Cao Y.X. (2007). Human Umbilical Cord Blood Serum in Culture Medium on Oocyte Maturation In Vitro. Arch. Androl..

[120] Giannaccare G., Carnevali A., Senni C., Logozzo L., Scorcia V. (2020). Umbilical Cord Blood and Serum for the Treatment of Ocular Diseases: A Comprehensive Review. Ophthalmol. Ther..

[121] Tovar A.A., White I.A., Sabater A.L. (2021). Use of Acellular Umbilical Cord-Derived Tissues in Corneal and Ocular Surface Diseases. Medicines.

[122] Versura P., Profazio V., Buzzi M., Stancari A., Arpinati M., Malavolta N., Campos E.C. (2013). Efficacy of Standardized and Quality-Controlled Cord Blood Serum Eye Drop Therapy in the Healing of Severe Corneal Epithelial Damage in Dry Eye. Cornea.

[123] Vajpayee R.B., Mukerji N., Tandon R., Sharma N., Pandey R.M., Biswas N.R., Malhotra N., Melki S.A. (2003). Evaluation of Umbilical Cord Serum Therapy for Persistent Corneal Epithelial Defects. Br. J. Ophthalmol..

[124] Yoon K.C., Im S.K., Park Y.G., Jung Y.D., Yang S.Y., Choi J. (2006). Application of Umbilical Cord Serum Eyedrops for the Treatment of Dry Eye Syndrome. Cornea.

[125] Oh H.J., Jang J.Y., Li Z., Park S.H., Yoon K.C. (2012). Effects of Umbilical Cord Serum Eye Drops in a Mouse Model of Ocular Chemical Burn. Curr. Eye Res..

[126] Subiran C., Kristensen S.G., Andersen C.Y. (2021). Umbilical Cord Blood–Derived Platelet-Rich Plasma: A Clinically Acceptable Substitute for Fetal Bovine Serum?. Fertil. Steril..

[127] de Miguel–Gómez L., López-Martínez S., Campo H., Francés-Herrero E., Faus A., Díaz A., Pellicer A., Domínguez F., Cervelló I. (2021). Comparison of Different Sources of Platelet-Rich Plasma as Treatment Option for Infertility-Causing Endometrial Pathologies. Fertil. Steril..

[128] Jain N.K., Gulati M. (2016). Platelet-Rich Plasma: A Healing Virtuoso. Blood Res..

[129] Tibial Fracture—Platelet-Rich Plasma and Bone Marrow Concentrate—Full Text View—ClinicalTrials.Gov. https://clinicaltrials.gov/ct2/show/NCT03100695.

[130] Gentile P., Garcovich S. (2020). Autologous Activated Platelet-Rich Plasma (AA-PRP) and Non-Activated (A-PRP) in Hair Growth: A Retrospective, Blinded, Randomized Evaluation in Androgenetic Alopecia. Expert. Opin. Biol. Ther..

[131] Use of Platelet-Rich Plasma (PRP) and Platelet-Poor Plasma (PPP) to Prevent Infection and Delayed Wound Healing—Full Text View—ClinicalTrials.Gov. https://clinicaltrials.gov/ct2/show/NCT01639144.

[132] Dório M., Pereira R.M.R., Luz A.G.B., Deveza L.A., de Oliveira R.M., Fuller R. (2021). Efficacy of Platelet-Rich Plasma and Plasma for Symptomatic Treatment of Knee Osteoarthritis: A Double-Blinded Placebo-Controlled Randomized Clinical Trial. BMC Musculoskelet. Disord..

[133] Sirithanabadeekul P., Dannarongchai A., Suwanchinda A. (2020). Platelet-Rich Plasma Treatment for Melasma: A Pilot Study. J. Cosmet. Dermatol..

[134] Ahmed M., Reffat S.A., Hassan A., Eskander F. (2017). Platelet-Rich Plasma for the Treatment of Clean Diabetic Foot Ulcers. Ann. Vasc. Surg..

[135] Schlüssel M.M., Keene D.J., Wagland S., Alsousou J., Lamb S.E., Willett K., Dutton S.J., Willett K., Alsousou J., Lamb S.E. (2018). Platelet-Rich Plasma in Achilles Tendon Healing 2 (PATH-2) Trial: Statistical Analysis Plan for a Multicentre, Double-Blinded, Parallel-Group, Placebo-Controlled Randomised Clinical Trial. Trials.

[136] Alam M., Hughart R., Champlain A., Geisler A., Paghdal K., Whiting D., Hammel J.A., Maisel A., Rapcan M.J., West D.P. (2018). Effect of Platelet-Rich Plasma Injection for Rejuvenation of Photoaged Facial Skin: A Randomized Clinical Trial. JAMA Dermatol..

[137] Caiaffa V., Ippolito F., Abate A., Nappi V., Santodirocco M., Visceglie D. (2021). Allogenic Platelet Concentrates from Umbilical Cord Blood for Knee Osteoarthritis: Preliminary Results. Med. Glas. (Zenica).

[138] Umbilical Cord Plasma for Treating Endometrial Pathologies (Thin Endometrium/Asherman’s Syndrome/Endometria Atrophy)—Full Text View—ClinicalTrials.Gov. https://clinicaltrials.gov/ct2/show/NCT05095597.

[139] Behroozi Z., Ramezani F., Janzadeh A., Rahimi B., Nasirinezhad F. (2021). Platelet-Rich Plasma in Umbilical Cord Blood Reduces Neuropathic Pain in Spinal Cord Injury by Altering the Expression of ATP Receptors: PRP, a New Window for Pain Reduction Post Spinal Cord Injury. Physiol. Behav..

[140] Guo D., Murdoch C.E., Liu T., Qu J., Jiao S., Wang Y., Wang W., Chen X. (2018). Therapeutic Angiogenesis of Chinese Herbal Medicines in Ischemic Heart Disease: A Review. Front. Pharmacol..

[141] Luo W., Gong Y., Qiu F., Yuan Y., Jia W., Liu Z., Gao L. (2021). NGF Nanoparticles Enhance the Potency of Transplanted Human Umbilical Cord Mesenchymal Stem Cells for Myocardial Repair. Am. J. Physiol. Heart Circ. Physiol..

[142] WenBo W., Fei Z., YiHeng D., Wei W., TingMang Y., WenHao Z., QianRu L., HaiTao L. (2017). Human Umbilical Cord Mesenchymal Stem Cells Overexpressing Nerve Growth Factor Ameliorate Diabetic Cystopathy in Rats. Neurochem. Res..

[143] Pan Y., Jiao G., Yang J., Guo R., Li J., Wang C. (2017). Insights into the Therapeutic Potential of Heparinized Collagen Scaffolds Loading Human Umbilical Cord Mesenchymal Stem Cells and Nerve Growth Factor for the Repair of Recurrent Laryngeal Nerve Injury. Tissue Eng. Regen. Med..

[144] Rafieemehr H., KheIrandish M., Soleimani M. (2015). Neuroprotective Effects of Transplanted Mesenchymal Stromal Cells-Derived Human Umbilical Cord Blood Neural Progenitor Cells in EAE. Iran J. Allergy Asthma Immunol..

[145] Jung N., Kong T.H., Yu Y., Park H., Lee E., Yoo S.M., Baek S.Y., Lee S., Kang K.S. (2022). Immunomodulatory Effect of Epidermal Growth Factor Secreted by Human Umbilical Cord Blood-Derived Mesenchymal Stem Cells on Atopic Dermatitis. Int. J. Stem Cells.

[146] Yan L., Zhou L., Yan B., Zhang L., Du W., Liu F., Yuan Q., Tong P., Shan L., Efferth T. (2020). Growth Factors-Based Beneficial Effects of Platelet Lysate on Umbilical Cord-Derived Stem Cells and Their Synergistic Use in Osteoarthritis Treatment. Cell Death Dis.

[147] Çil N., Oğuz E.O., Mete E., Çetinkaya A., Mete G. (2017). Effects of Umbilical Cord Blood Stem Cells on Healing Factors for Diabetic Foot Injuries. Biotech. Histochem..

[148] Rizvanov A.A., Guseva D.S., Salafutdinov I.I., Kudryashova N.V., Bashirov F.V., Kiyasov A.P., Yalvaç M.E., Gazizov I.M., Kaligin M.S., Sahin F. (2011). Genetically Modified Human Umbilical Cord Blood Cells Expressing Vascular Endothelial Growth Factor and Fibroblast Growth Factor 2 Differentiate into Glial Cells after Transplantation into Amyotrophic Lateral Sclerosis Transgenic Mice. Exp. Biol. Med. (Maywood).

[149] Mao C., Hou X., Wang B., Chi J., Jiang Y., Zhang C., Li Z. (2017). Intramuscular Injection of Human Umbilical Cord-Derived Mesenchymal Stem Cells Improves Cardiac Function in Dilated Cardiomyopathy Rats. Stem Cell Res. Ther..

[150] Yin F., Wang W.Y., Mao L.C., Cai Q.Q., Jiang W.H. (2020). Effect of Human Umbilical Cord Mesenchymal Stem Cells Transfected with HGF on TGF-Β1/Smad Signaling Pathway in Carbon Tetrachloride-Induced Liver Fibrosis Rats. Stem Cells Dev..

[151] He Y., Guo X., Lan T., Xia J., Wang J., Li B., Peng C., Chen Y., Hu X., Meng Z. (2021). Human Umbilical Cord-Derived Mesenchymal Stem Cells Improve the Function of Liver in Rats with Acute-on-Chronic Liver Failure via Downregulating Notch and Stat1/Stat3 Signaling. Stem Cell Res. Ther..

[152] Liu X.S., Li J.F., Wang S.S., Wang Y.T., Zhang Y.Z., Yin H.L., Geng S., Gong H.C., Han B., Wang Y.L. (2014). Human Umbilical Cord Mesenchymal Stem Cells Infected with Adenovirus Expressing HGF Promote Regeneration of Damaged Neuron Cells in a Parkinson’s Disease Model. Biomed Res. Int..

[153] Chen H., Tang S., Liao J., Liu M., Lin Y. (2019). Therapeutic Effect of Human Umbilical Cord Blood Mesenchymal Stem Cells Combined with G-CSF on Rats with Acute Liver Failure. Biochem. Biophys. Res. Commun..

[154] Acosta S.A., Tajiri N., Shinozuka K., Ishikawa H., Sanberg P.R., Sanchez-Ramos J., Song S., Kaneko Y., Borlongan C.V. (2014). Combination Therapy of Human Umbilical Cord Blood Cells and Granulocyte Colony Stimulating Factor Reduces Histopathological and Motor Impairments in an Experimental Model of Chronic Traumatic Brain Injury. PLoS ONE.

[155] Eftekhar M., Naghshineh E., Khani P. (2018). Role of Granulocyte Colony-Stimulating Factor in Human Reproduction. J. Res. Med. Sci..

[156] Fleetwood A.J., Achuthan A., Hamilton J.A. (2016). Colony Stimulating Factors (CSFs). Encycl. Immunobiol..

[157] Zhu H., Xiong Y., Xia Y., Zhang R., Tian D., Wang T., Dai J., Wang L., Yao H., Jiang H. (2017). Therapeutic Effects of Human Umbilical Cord-Derived Mesenchymal Stem Cells in Acute Lung Injury Mice. Sci. Rep..

[158] Wang H., Wen Y., Polan M.L., Boostanfar R., Feinman M., Behr B. (2005). Exogenous Granulocyte–Macrophage Colony-Stimulating Factor Promotes Follicular Development in the Newborn Rat in Vivo. Hum. Reprod..

[159] Zhang R., Yin L., Zhang B., Shi H., Sun Y., Ji C., Chen J., Wu P., Zhang L., Xu W. (2018). Resveratrol Improves Human Umbilical Cord-Derived Mesenchymal Stem Cells Repair for Cisplatin-Induced Acute Kidney Injury. Cell Death Dis..

[160] Zhang X., Gao F., Yan Y., Ruan Z., Liu Z. (2015). Combination Therapy with Human Umbilical Cord Mesenchymal Stem Cells and Angiotensin-Converting Enzyme 2 Is Superior for the Treatment of Acute Lung Ischemia-Reperfusion Injury in Rats. Cell Biochem. Funct..

[161] Shrestha C., Zhao L., Chen K., He H., Mo Z. (2013). Enhanced Healing of Diabetic Wounds by Subcutaneous Administration of Human Umbilical Cord Derived Stem Cells and Their Conditioned Media. Int. J. Endocrinol..

[162] Sungkar T., Putra A., Lindarto D., Juwita Sembiring R. (2021). Anti-Fibrotic Effect of Intravenous Umbilical Cord-Derived Mesenchymal Stem Cells (UC-MSCs) Injection in Experimental Rats Induced Liver Fibrosis. Med. Glas. (Zenica).

[163] Guérit E., Arts F., Dachy G., Boulouadnine B., Demoulin J.B. (2021). PDGF receptor mutations in human diseases. Cell. Mol. Life Sci..

[164] Park H.H., Lee S., Yu Y., Yoo S.M., Baek S.Y., Jung N., Seo K.W., Kang K.S. (2020). TGF-β Secreted by Human Umbilical Cord Blood-Derived Mesenchymal Stem Cells Ameliorates Atopic Dermatitis by Inhibiting Secretion of TNF-α and IgE. Stem Cells.

[165] Zhang Y., Pan Y., Liu Y., Li X., Tang L., Duan M., Li J., Zhang G. (2021). Exosomes Derived from Human Umbilical Cord Blood Mesenchymal Stem Cells Stimulate Regenerative Wound Healing via Transforming Growth Factor-β Receptor Inhibition. Stem Cell Res. Ther..

[166] Yu Y., Hu D., Zhou Y., Xiang H., Liu B., Shen L., Long C., Liu X., Lin T., He D. (2020). Human Umbilical Cord Mesenchymal Stem Cell Attenuates Renal Fibrosis via TGF-β/Smad Signaling Pathways in Vivo and in Vitro. Eur. J. Pharmacol..

[167] Li D., Liu Q., Qi L., Dai X., Liu H., Wang Y. (2016). Low Levels of TGF-Β1 Enhance Human Umbilical Cord-Derived Mesenchymal Stem Cell Fibronectin Production and Extend Survival Time in a Rat Model of Lipopolysaccharide-Induced Acute Lung Injury. Mol. Med. Rep..

[168] Khoshakhlagh M., Soleimani A., Binabaj M.M., Avan A., Ferns G.A., Khazaei M., Hassanian S.M. (2019). Therapeutic Potential of Pharmacological TGF-β Signaling Pathway Inhibitors in the Pathogenesis of Breast Cancer. Biochem. Pharmacol..

[169] Zhou X., Gu J., Gu Y., He M., Bi Y., Chen J., Li T. (2015). Human Umbilical Cord-Derived Mesenchymal Stem Cells Improve Learning and Memory Function in Hypoxic-Ischemic Brain-Damaged Rats via an IL-8-Mediated Secretion Mechanism Rather than Differentiation Pattern Induction. Cell Physiol. Biochem..

[170] Huang Z.F., Zhu J., Lu S.H., Zhang J.L., Chen X.D., Du L.X., Yang Z.G., Song Y.K., Wu D.Y., Liu B. (2013). Inhibitory Effect of Human Umbilical Cord-Derived Mesenchymal Stem Cells on Interleukin-17 Production in Peripheral Blood T Cells from Spondyloarthritis Patients. Zhongguo Shi Yan Xue Ye Xue Za Zhi.

[171] Kim Y.J., Ahn H.J., Lee S.H., Lee M.H., Kang K.S. (2020). Effects of Conditioned Media from Human Umbilical Cord Blood-Derived Mesenchymal Stem Cells in the Skin Immune Response. Biomed Pharmacother..

[172] Zhao W.H., Cheng J.X., Shi P.F., Huang J.Y. (2011). Human Umbilical Cord Mesenchymal Stem Cells with Adenovirus-Mediated Interleukin 12 Gene Transduction Inhibits the Growth of Ovarian Carcinoma Cells Both in Vitro and in Vivo. Nan Fang Yi Ke Da Xue Xue Bao.

[173] Niu X., Xu X., Luo Z., Wu D., Tang J. (2020). The Expression of Th9 and Th22 Cells in Rats with Cerebral Palsy after HUC-MSC Transplantation. J. Chin. Med. Assoc..

[174] Liu M., He J., Zheng S., Zhang K., Ouyang Y., Zhang Y., Li C., Wu D. (2021). Human Umbilical Cord Mesenchymal Stem Cells Ameliorate Acute Liver Failure by Inhibiting Apoptosis, Inflammation and Pyroptosis. Ann. Transl. Med..

[175] Zhang C., Zhu Y., Wang J., Hou L., Li W., An H. (2019). CXCR4-Overexpressing Umbilical Cord Mesenchymal Stem Cells Enhance Protection against Radiation-Induced Lung Injury. Stem Cells Int..

[176] Ma J., Liu N., Yi B., Zhang X., Gao B.B., Zhang Y., Xu R., Li X., Dai Y. (2015). Transplanted HUCB-MSCs Migrated to the Damaged Area by SDF-1/CXCR4 Signaling to Promote Functional Recovery after Traumatic Brain Injury in Rats. Neurol Res..

[177] Liu Y., Cai J., Luo X., Wen H., Luo Y. (2020). Collagen Scaffold with Human Umbilical Cord Mesenchymal Stem Cells Remarkably Improves Intrauterine Adhesions in a Rat Model. Gynecol. Obstet. Invest..

[178] Borish L.C., Steinke J.W. (2003). Cytokines and chemokines. J. Allergy Clin. Immunol..

[179] Na J., Kim G.J. (2020). Recent Trends in Stem Cell Therapy for Premature Ovarian Insufficiency and Its Therapeutic Potential: A Review. J. Ovarian Res..

[180] Li J., Mao Q.X., He J.J., She H.Q., Zhang Z., Yin C.Y. (2017). Human Umbilical Cord Mesenchymal Stem Cells Improve the Reserve Function of Perimenopausal Ovary via a Paracrine Mechanism. Stem Cell Res. Ther..

[181] Zhao Y., Ma J., Yi P., Wu J., Zhao F., Tu W., Liu W., Li T., Deng Y., Hao J. (2020). Human Umbilical Cord Mesenchymal Stem Cells Restore the Ovarian Metabolome and Rescue Premature Ovarian Insufficiency in Mice. Stem Cell Res. Ther..

[182] Lu X., Bao H., Cui L., Zhu W., Zhang L., Xu Z., Man X., Chu Y., Fu Q., Zhang H. (2020). HUMSC Transplantation Restores Ovarian Function in POI Rats by Inhibiting Autophagy of Theca-Interstitial Cells via the AMPK/MTOR Signaling Pathway. Stem Cell Res. Ther..

[183] Shen J., Cao D., Sun J.-L. (2020). Ability of Human Umbilical Cord Mesenchymal Stem Cells to Repair Chemotherapy-Induced Premature Ovarian Failure. World J. Stem Cells.

[184] Jalalie L., Rezaie M.J., Jalili A., Rezaee M.A., Vahabzadeh Z., Rahmani M.R., Karimipoor M., Hakhamaneshi M.S. (2019). Distribution of the CM-Dil-Labeled Human Umbilical Cord Vein Mesenchymal Stem Cells Migrated to the Cyclophosphamide-Injured Ovaries in C57BL/6 Mice. Iran Biomed J..

[185] Zhang L., Sun Y., Zhang X.-X., Liu Y.-B., Sun H.-Y., Wu C.-T., Xiao F.-J., Wang L.-S. (2022). Comparison of CD146 +/− Mesenchymal Stem Cells in Improving Premature Ovarian Failure. Stem Cell Res. Ther..

[186] Wang Z., Wei Q., Wang H., Han L., Dai H., Qian X., Yu H., Yin M., Shi F., Qi N. (2020). Mesenchymal Stem Cell Therapy Using Human Umbilical Cord in a Rat Model of Autoimmune-Induced Premature Ovarian Failure. Stem Cells Int..

[187] Lv X., Guan C., Li Y., Su X., Zhang L., Wang X., Xia H.F., Ma X. (2021). Effects of Single and Multiple Transplantations of Human Umbilical Cord Mesenchymal Stem Cells on the Recovery of Ovarian Function in the Treatment of Premature Ovarian Failure in Mice. J. Ovarian Res..

[188] Shi L., Zhang Y., Dong X., Pan Y., Ying H., Chen J., Yang W., Zhang Y., Fei H., Liu X. (2022). Toxicity from a Single Injection of Human Umbilical Cord Mesenchymal Stem Cells into Rat Ovaries. Reprod. Toxicol..

[189] Pan Y., Zhang L., Zhang X., Hu C., Liu R. (2016). Biological and Biomechanical Analysis of Two Types of Mesenchymal Stem Cells for Intervention in Chemotherapy-Induced Ovarian Dysfunction. Arch. Gynecol. Obstet..

[190] Cui L., Bao H., Liu Z., Man X., Liu H., Hou Y., Luo Q., Wang S., Fu Q., Zhang H. (2020). HUMSCs Regulate the Differentiation of Ovarian Stromal Cells via TGF-Β1/Smad3 Signaling Pathway to Inhibit Ovarian Fibrosis to Repair Ovarian Function in POI Rats. Stem Cell Res. Ther..

[191] Zhang M., Xie T., Dai W., Zhao B., Zheng Y., Hu J., Pan R., Wang L. (2022). Umbilical Cord Mesenchymal Stem Cells Ameliorate Premature Ovarian Insufficiency in Rats. Evid.-Based Complementary Altern. Med..

[192] Zhang J., Xiong J., Fang L., Lu Z., Wu M., Shi L., Qin X., Luo A., Wang S. (2016). The Protective Effects of Human Umbilical Cord Mesenchymal Stem Cells on Damaged Ovarian Function: A Comparative Study. Biosci. Trends.

[193] Yan L., Wu Y., Li L., Wu J., Zhao F., Gao Z., Liu W., Li T., Fan Y., Hao J. (2020). Clinical Analysis of Human Umbilical Cord Mesenchymal Stem Cell Allotransplantation in Patients with Premature Ovarian Insufficiency. Cell Prolif..

[194] Lu X., Cui J., Cui L., Luo Q., Cao Q., Yuan W., Zhang H. (2019). The Effects of Human Umbilical Cord-Derived Mesenchymal Stem Cell Transplantation on Endometrial Receptivity Are Associated with Th1/Th2 Balance Change and UNK Cell Expression of Uterine in Autoimmune Premature Ovarian Failure Mice. Stem Cell Res. Ther..

[195] Ramathal C.Y., Bagchi I.C., Taylor R.N., Bagchi M.K. (2010). Endometrial Decidualization: Of Mice and Men. Semin. Reprod. Med..

[196] Aygün E.G., Tümentemur G. (2022). Effects of Stem Cells and Amniotic Fluid on Uterus and Ovaries on a Rat Model with Abdominal Adhesions: A Controlled Study. J. Turk. Ger. Gynecol. Assoc..

[197] Jiao W., Mi X., Yang Y., Liu R., Liu Q., Yan T., Chen Z.J., Qin Y., Zhao S. (2022). Mesenchymal Stem Cells Combined with Autocrosslinked Hyaluronic Acid Improve Mouse Ovarian Function by Activating the PI3K-AKT Pathway in a Paracrine Manner. Stem Cell Res. Ther..

[198] Mi X., Jiao W., Yang Y., Qin Y., Chen Z.J., Zhao S. (2022). HGF Secreted by Mesenchymal Stromal Cells Promotes Primordial Follicle Activation by Increasing the Activity of the PI3K-AKT Signaling Pathway. Stem Cell Rev. Rep..

[199] Francés-Herrero E., Lopez R., Hellström M., de Miguel-Gómez L., Herraiz S., Brännström M., Pellicer A., Cervelló I. (2022). Bioengineering Trends in Female Reproduction: A Systematic Review. Hum. Reprod. Update.

[200] Ding L., Yan G., Wang B., Xu L., Gu Y., Ru T., Cui X., Lei L., Liu J., Sheng X. (2018). Transplantation of UC-MSCs on Collagen Scaffold Activates Follicles in Dormant Ovaries of POF Patients with Long History of Infertility. Sci. China Life Sci..

[201] Yang Y., Lei L., Wang S., Sheng X., Yan G., Xu L., Liu J., Liu M., Zhen X., Ding L. (2019). Transplantation of Umbilical Cord–Derived Mesenchymal Stem Cells on a Collagen Scaffold Improves Ovarian Function in a Premature Ovarian Failure Model of Mice. In Vitro Cell Dev. Biol. Anim..

[202] Liu F., Hu S., Yang H., Li Z., Huang K., Su T., Wang S. (2019). Hyaluronic Acid Hydrogel Integrated with Mesenchymal Stem Cell-Secretome to Treat Endometrial Injury in a Rat Model of Asherman’s Syndrome. Adv. Healthc. Mater..

[203] Yaghoubi Y., Movassaghpour A.A., Zamani M., Talebi M., Mehdizadeh A., Yousefi M. (2019). Human Umbilical Cord Mesenchymal Stem Cells Derived-Exosomes in Diseases Treatment. Life Sci..

[204] Liu C., Yin H., Jiang H., Du X., Wang C., Liu Y., Li Y., Yang Z. (2020). Extracellular Vesicles Derived from Mesenchymal Stem Cells Recover Fertility of Premature Ovarian Insufficiency Mice and the Effects on Their Offspring. Cell Transplant..

[205] Li Z., Zhang M., Zheng J., Tian Y., Zhang H., Tan Y., Li Q., Zhang J., Huang X. (2021). Human Umbilical Cord Mesenchymal Stem Cell-Derived Exosomes Improve Ovarian Function and Proliferation of Premature Ovarian Insufficiency by Regulating the Hippo Signaling Pathway. Front. Endocrinol. (Lausanne).

[206] Gershon E., Dekel N. (2020). Newly Identified Regulators of Ovarian Folliculogenesis and Ovulation. Int. J. Mol. Sci..

[207] Velarde F., Castañeda V., Morales E., Ortega M., Ocaña E., Álvarez-Barreto J., Grunauer M., Eguiguren L., Caicedo A. (2020). Use of Human Umbilical Cord and Its Byproducts in Tissue Regeneration. Front. Bioeng. Biotechnol..

[208] Braunstein S., Kaplan G., Gottlieb A.B., Schwartz M., Walshs G., Abalos R.M., Fajardo T.T., Guido L.S., Krueger J.G. (1994). GM-CSF Activates Regenerative Epidermal Growth and Stimulates Keratinocyte Proliferation in Human Skin in Vivo. J. Investig. Dermatol..

[209] Zhang J., Yan L., Wang Y., Zhang S., Xu X., Dai Y., Zhao S., Li Z., Zhang Y., Xia G. (2020). In Vivo and in Vitro Activation of Dormant Primordial Follicles by EGF Treatment in Mouse and Human. Clin. Transl. Med..

[210] Wang J., Zhao Y., Zheng F., Ma N., Qin R., Qin W., Liu B., Qin A. (2021). Activated Human Umbilical Cord Blood Platelet-Rich Plasma Enhances the Beneficial Effects of Human Umbilical Cord Mesenchymal Stem Cells in Chemotherapy-Induced POF Rats. Stem Cells Int..

[211] de Miguel-Gómez L., López-Martínez S., Francés-Herrero E., Rodríguez-Eguren A., Pellicer A., Cervelló I. (2021). Stem Cells and the Endometrium: From the Discovery of Adult Stem Cells to Pre-Clinical Models. Cells.

[212] Santamaria X., Cabanillas S., Cervelló I., Arbona C., Raga F., Ferro J., Palmero J., Remohí J., Pellicer A., Simón C. (2016). Autologous Cell Therapy with CD133+ Bone Marrow-Derived Stem Cells for Refractory Asherman’s Syndrome and Endometrial Atrophy: A Pilot Cohort Study. Hum. Reprod..

[213] Tan J., Li P., Wang Q., Li Y., Li X., Zhao D., Xu X., Kong L. (2016). Autologous Menstrual Blood-Derived Stromal Cells Transplantation for Severe Asherman’s Syndrome. Hum. Reprod..

[214] Zheng J.H., Zhang J.K., Kong D.S., Song Y.B., Zhao S.D., Qi W.B., Li Y.N., Zhang M.L., Huang X.H. (2020). Quantification of the CM-Dil-Labeled Human Umbilical Cord Mesenchymal Stem Cells Migrated to the Dual Injured Uterus in SD Rat. Stem Cell Res. Ther..

[215] Zhang L., Li Y., Dong Y.C., Guan C.Y., Tian S., Lv X.D., Li J.H., Su X., Xia H.F., Ma X. (2022). Transplantation of Umbilical Cord-Derived Mesenchymal Stem Cells Promotes the Recovery of Thin Endometrium in Rats. Sci. Rep..

[216] Zhuang M., Zhang W., Cheng N., Zhou L., Liu D., Yan H., Fang G., Heng B.C., Sun Y., Tong G. (2022). Human Umbilical Cord Mesenchymal Stromal Cells Promote the Regeneration of Severe Endometrial Damage in a Rat Model. Acta Biochim. Biophys. Sin. (Shanghai).

[217] Cao Y., Sun H., Zhu H., Zhu X., Tang X., Yan G., Wang J., Bai D., Wang J., Wang L. (2018). Allogeneic Cell Therapy Using Umbilical Cord MSCs on Collagen Scaffolds for Patients with Recurrent Uterine Adhesion: A Phase i Clinical Trial. Stem Cell Res. Ther..

[218] Wang S., Shi C., Cai X., Wang Y., Chen X., Han H., Shen H. (2021). Human Acellular Amniotic Matrix with Previously Seeded Umbilical Cord Mesenchymal Stem Cells Restores Endometrial Function in a Rat Model of Injury. Mediators Inflamm..

[219] Zhou S., Lei Y., Wang P., Chen J., Zeng L., Qu T., Maldonado M., Huang J., Han T., Wen Z. (2022). Human Umbilical Cord Mesenchymal Stem Cells Encapsulated with Pluronic F-127 Enhance the Regeneration and Angiogenesis of Thin Endometrium in Rat via Local IL-1β Stimulation. Stem Cells Int..

[220] Wang L., Yu C., Chang T., Zhang M., Song S., Xiong C., Su P., Xiang W. (2020). In Situ Repair Abilities of Human Umbilical Cord-Derived Mesenchymal Stem Cells and Autocrosslinked Hyaluronic Acid Gel Complex in Rhesus Monkeys with Intrauterine Adhesion. Sci. Adv..

[221] Krenning G., Harmsen M.C. (2015). MicroRNAs in Tissue Engineering and Regenerative Medicine. MicroRNA in Regenerative Medicine.

[222] Singh R., Kaundal R.K., Zhao B., Bouchareb R., Lebeche D. (2021). Resistin Induces Cardiac Fibroblast-Myofibroblast Differentiation through JAK/STAT3 and JNK/c-Jun Signaling. Pharmacol. Res..

[223] Liu J., Shang B., Bai J. (2020). IL-22/IL-22R1 Promotes Proliferation and Collagen Synthesis of MRC-5 Cells via the JAK/STAT3 Signaling Pathway and Regulates Airway Subepithelial Fibrosis. Exp. Ther. Med..

[224] Yang L., Han B., Zhang M., Wang Y.H., Tao K., Zhu M.X., He K., Zhang Z.G., Hou S. (2020). Activation of BK Channels Prevents Hepatic Stellate Cell Activation and Liver Fibrosis Through the Suppression of TGFβ1/SMAD3 and JAK/STAT3 Profibrotic Signaling Pathways. Front. Pharmacol..

[225] Zhu D., Cheng K. (2021). Cardiac Cell Therapy for Heart Repair: Should the Cells Be Left Out?. Cells.

[226] Ebrahim N., Mostafa O., el Dosoky R.E., Ahmed I.A., Saad A.S., Mostafa A., Sabry D., Ibrahim K.A., Farid A.S. (2018). Human Mesenchymal Stem Cell-Derived Extracellular Vesicles/Estrogen Combined Therapy Safely Ameliorates Experimentally Induced Intrauterine Adhesions in a Female Rat Model. Stem Cell Res. Ther..

[227] Cai Y., Wu F., Yu Y., Liu Y., Shao C., Gu H., Li M., Zhao Y. (2019). Porous Scaffolds from Droplet Microfluidics for Prevention of Intrauterine Adhesion. Acta Biomater..

[228] Li X., Sun H., Lin N., Hou X., Wang J., Zhou B., Xu P., Xiao Z., Chen B., Dai J. (2011). Regeneration of Uterine Horns in Rats by Collagen Scaffolds Loaded with Collagen-Binding Human Basic Fibroblast Growth Factor. Biomaterials.

[229] Jiang P., Tang X., Wang H., Dai C., Su J., Zhu H., Song M., Liu J., Nan Z., Ru T. (2019). Collagen-Binding Basic Fibroblast Growth Factor Improves Functional Remodeling of Scarred Endometrium in Uterine Infertile Women: A Pilot Study. Sci. China Life Sci..

[230] López-Martínez S., Rodríguez-Eguren A., de Miguel-Gómez L., Francés-Herrero E., Faus A., Díaz A., Pellicer A., Ferrero H., Cervelló I. (2021). Bioengineered Endometrial Hydrogels with Growth Factors Promote Tissue Regeneration and Restore Fertility in Murine Models. Acta Biomater..

[231] Rodríguez-Eguren A., de Miguel-Gómez L., Francés-Herrero E., Gómez-Álvarez M., Faus A., Gómez-Cerdá M., Moret-Tatay I., Díaz A., Pellicer A., Cervelló I. (2022). Human Umbilical Cord Platelet-Rich Plasma to Treat Endometrial Pathologies: Methodology, Composition and Pre-Clinical Models. Hum. Reprod. Open.

[232] Zheng S., Gao Y., Chen K., Liu Y., Xia N., Fang F. (2022). A Robust and Highly Efficient Approach for Isolation of Mesenchymal Stem Cells from Wharton’s Jelly for Tissue Repair. Cell Transplant..

[233] Hamahata Y., Akagi K., Maeda T., Nemoto K., Koike J. (2022). Management of Pelvic Organ Prolapse (POP) and Rectal Prolapse. J. Anus. Rectum. Colon..

[234] Cheng J., Zhao Z.W., Wen J.R., Wang L., Huang L.W., Yang Y.L., Zhao F.N., Xiao J.Y., Fang F., Wu J. (2020). Status, Challenges, and Future Prospects of Stem Cell Therapy in Pelvic Floor Disorders. World J. Clin. Cases.

[235] Ma Y., Zhang Y., Chen J., Li L., Liu X., Zhang L., Ma C., Wang Y., Tian W., Song X. (2021). Mesenchymal Stem Cell-Based Bioengineered Constructs Enhance Vaginal Repair in Ovariectomized Rhesus Monkeys. Biomaterials.

[236] Mao M., Li Y., Zhang Y., Kang J., Zhu L. (2021). Human Umbilical Cord Mesenchymal Stem Cells Reconstruct the Vaginal Wall of Ovariectomized Sprague–Dawley Rats: Implications for Pelvic Floor Reconstruction. Cell Tissue Res..

[237] Deng M., Ding J., Ai F., Mao M., Zhu L. (2020). Impact of Human Umbilical Cord–Derived Stem Cells (HUMSCs) on Host Responses to a Synthetic Polypropylene Mesh for Pelvic Floor Reconstruction in a Rat Model. Cell Tissue Res..

[238] Norton P., Brubaker L. (2006). Urinary Incontinence in Women. Lancet.

[239] Hannestad Y.S., Rortveit G., Sandvik H., Hunskaar S. (2000). A Community-Based Epidemiological Survey of Female Urinary Incontinence: The Norwegian EPINCONT Study. J. Clin. Epidemiol..

[240] Lee C.N., Jang J.B., Kim J.Y., Koh C., Baek J.Y., Lee K.J. (2010). Human Cord Blood Stem Cell Therapy for Treatment of Stress Urinary Incontinence. J. Korean Med. Sci..

[241] Liao W., Tang X., Li X., Li T. (2019). Therapeutic Effect of Human Umbilical Cord Mesenchymal Stem Cells on Tubal Factor Infertility Using a Chronic Salpingitis Murine Model. Arch. Gynecol. Obstet..

[242] Detels R., Green A.M., Klausner J.D., Katzenstein D., Gaydos C., Handsfield H.H., Pequegnat W., Mayer K., Hartwell T.D., Quinn T.C. (2011). The Incidence and Correlates of Symptomatic and Asymptomatic Chlamydia Trachomatis and Neisseria Gonorrhoeae Infections in Selected Populations in Five Countries. Sex Transm. Dis..

[243] Griebel C.P., Halvorsen J., Golemon T.B., Day A.A. (2005). Management of Spontaneous Abortion. Am. Fam. Physician.

[244] Chen X., Yang X., Wu R., Chen W., Xie H., Qian X., Zhang Y. (2016). Therapeutic Effects of Wharton Jelly-Derived Mesenchymal Stem Cells on Rat Abortion Models. J. Obstet. Gynaecol. Res..

[245] Xie Q., Liu R., Jiang J., Peng J., Yang C., Zhang W., Wang S., Song J. (2020). What Is the Impact of Human Umbilical Cord Mesenchymal Stem Cell Transplantation on Clinical Treatment?. Stem Cell Res. Ther..

[246] Baba K., Yamazaki Y., Sone Y., Sugimoto Y., Moriyama K., Sugimoto T., Kumazawa K., Shimakura Y., Takeda A. (2019). An in Vitro Long-Term Study of Cryopreserved Umbilical Cord Blood-Derived Platelet-Rich Plasma Containing Growth Factors—PDGF-BB, TGF-β and VEGF. J. Cranio-Maxillofac. Surg..

[247] Francés-Herrero E., Rodríguez-Eguren A., Gómez-Álvarez M., Miguel-Gómez L.d., Ferrero H., Cervelló I. (2022). Future Challenges and Opportunities of Extracellular Matrix Hydrogels in Female Reproductive Medicine. Int. J. Mol. Sci..

[248] Center for Biologics Evaluation and Research BLA for Minimally Manipulated, Unrelated Allogeneic Placental/Umbilical Cord Blood Intended for Hematopoietic and Immunologic Reconstitution in Patients with Disorders Affecting the Hematopoietic System | FDA. https://www.fda.gov/regulatory-information/search-fda-guidance-documents/bla-minimally-manipulated-unrelated-allogeneic-placentalumbilical-cord-blood-intended-hematopoietic.

[249] The Efficacy and Safety of Collagen Scaffold Loaded with Umbilical Cord Derived Mesenchymal Stem Cells in Infertile Women with Thin Endometrium or Endometrial Scarring—Full Text View—ClinicalTrials.Gov. https://clinicaltrials.gov/ct2/show/NCT03592849?term=endometrium%2C+umbilical+cord&draw=2&rank=2.

[250] HUC Mesenchymal Stem Cells (19#iSCLife®-UT) Therapy for Patients with Thin Endometrial Infertility—Full Text View—ClinicalTrials.Gov. https://clinicaltrials.gov/ct2/show/NCT05495711?term=endometrium%2C+umbilical+cord&draw=2&rank=5.

[251] Clinical Study of Human Umbilical Cord Mesenchymal Stem Cells in the Treatment of Premature Ovarian Insufficiency—Full Text View—ClinicalTrials.Gov. https://clinicaltrials.gov/ct2/show/NCT05308342?term=ovary%2C+umbilical+cord&draw=2&rank=1.

[252] Stem Cell Therapy Combined Hormone Replacement Therapy in Patients with Premature Ovarian Failure—Full Text View—ClinicalTrials.Gov. https://clinicaltrials.gov/ct2/show/NCT01742533?term=ovary%2C+umbilical+cord&draw=2&rank=3.

[253] Stem Cells and Secretomes for Infertility Therapy in Polycystic Ovary Syndrome (PCOS) Patients with Insulin Resistance.—Full Text View—ClinicalTrials.Gov. https://clinicaltrials.gov/ct2/show/NCT05279768?term=umbilical+cord+stem+cell&cond=ovary&draw=2&rank=1.

[254] Chen H., Tang Q.L., Wu X.Y., Xie L.C., Lin L.M., Ho G.Y., Ma L. (2015). Differentiation of Human Umbilical Cord Mesenchymal Stem Cells into Germ-like Cells in Mouse Seminiferous Tubules. Mol. Med. Rep..

[255] Abd Allah S.H., Pasha H.F., Abdelrahman A.A., Mazen N.F. (2017). Molecular Effect of Human Umbilical Cord Blood CD34-Positive and CD34-Negative Stem Cells and Their Conjugate in Azoospermic Mice. Mol. Cell Biochem..

[256] Hassan A.I., Alam S.S. (2014). Evaluation of Mesenchymal Stem Cells in Treatment of Infertility in Male Rats. Stem Cell Res. Ther..

[257] Tamadon A., Zhan-byrbekuly U., Kairgaliyev I., Khoradmehr A. (2019). Mesenchymal Stem Cell Therapy of Male Infertility. Male Reproductive Health.

