# Supplementary material for: Human Umbilical Cord-Based Therapeutics: Stem Cells and Blood Derivatives for Female Reproductive Medicine
Source: Int J Mol Sci. 2022 Dec 14;23(24):15942. doi: 10.3390/ijms232415942 (PMC9785531; doi:10.3390/ijms232415942)
Supplement: Supplementary file 1 [file ijms-23-15942-s001.zip › ijms-2037261-supplementary.pdf]

Supplementary Table S1. Ongoing hUC and derivatives application in human clinical trials.

TREATMENT	CONDITION	ADMINISTRATION	ANALIZED PARAMETERS	CT NUMBER	STATE	REFERENCE
hUC-PRP	IUA	Intrauterine injection (n=45)	Endometrial thickness, pregnancy rate, molecular regeneration	NCT05095597	Ongoing	[138]
hUC-MSC + collagen	IUA	Intrauterine instillation (n=20)	Endometrial thickness, pregnancy rate, live birth rate, menstrual blood volume, AE	NCT03592849	Ongoing	[251]
hUC-MSC + collagen	IUA	Intrauterine injection twice (n=24)	Pregnancy rate, intrauterine adhesions<br>Menstrual blood volume, endometrial receptivity	NCT05495711	Ongoing	[252]
hUC-MSC	POI	Intraovarian transplantation (n=66)	Follicular development rate, ovarian blood flow, pregnancy rate	NCT05308342	Ongoing	[253]
hUC-MSC	POI	Intraovarian transplantation	FSH level, uterine and ovarian characteristics, Kupperman Score,	NCT01742533	Unknown	[254]

		(n=40)	AE			
hUC-MSC	PCOS	Intravenous injection	Blood serum markers, free Androgen index, insulin resistance, Sex Hormone Binding Globulin and Anti-Mullerian Hormone concentration, Leptin and Adiponectine Profile, Cytokyne profile	NCT05279768	Ongoing	[255]

hUC-PRP, human umbilical cord platelet-rich plasma; hUC-MSC, human umbilical cord mesenchymal stem cells; IUA, intrauterine adhesions; POI, premature ovarian insufficiency; PCOS, polycystic ovarian syndrome; FSH, follicle-stimulating hormone; AE, adverse events.
